# A machine learning framework for predicting healthcare insurance claim denials

**DOI:** 10.3389/frhs.2026.1927175

**Published:** 2026-10-14

**Authors:** Muhammad Mohsin Jadoon, Obaidullah Miakhil, Noor Rahman Chakari, Muhammad Bilal Qureshi, Ihsan Ullah, Sami Ahmed Haider, Masood Ur Rehman

**Affiliations:** 1 School of Computing Sciences, Pak-Austria Fachhochschule: Institute of Applied Sciences and Technology, Haripur, Pakistan; 2 Department of Biomedical Engineering, Tohoku University, Sendai, Miyagi, Japan; 3 Computer Science Department, School of Engineering, Central Asian University, Tashkent, Uzbekistan; 4 Department of Electrical Engineering, COMSATS University Islamabad, Abbottabad, Pakistan; 5 Heriot-Watt International Faculty, K.Zhubanov Aktobe Regional University, Aktobe, Kazakhstan; 6 Electrical, Electronics and Computer Engineering Department, School of Engineering and Physical Sciences, Heriot Watt University, Edinburgh, United Kingdom; 7 James Watt School of Engineering, University of Glasgow, University Avenue, Glasgow, United Kingdom

**Keywords:** claim denial prediction, class imbalance, explainable artificial intelligence, health insurance claims, random forest, revenue cycle management

## Abstract

**Introduction:**

The denial of healthcare insurance claims imposes a significant burden on providers, leading to decreased revenue and inefficient operations. This research presents a predictive model to assist providers in managing claim submissions.

**Methods:**

The framework uses a rule-based claim scrubber that authenticates submissions before sending them, matching them against known denial patterns. It also employs a two-stage supervised pipeline. The study analyses 678,881 professional claim service lines submitted to a single U.S. payer between 2017 and 2024, of which 31.9% were denied. Stage 1 classifies a submitted line as accepted or denied using a Random Forest over 26 features

**Results:**

On a temporally ordered, claim-grouped held-out partition of 139,615 lines that retains the natural denial prevalence, it attains a precisionrecall AUC of 0.777 (95% CI 0.770-0.784) against a no-skill baseline of 0.330, ROC-AUC 0.865, accuracy 0.794, balanced accuracy 0.784, recall 0.756 and Matthews correlation 0.552. Stage 2 assigns a reason code over a 67-class label space covering 98.8% of denials, reaching 0.269 Top-1 and 0.498 Top-3 accuracy; the same model reaches 0.918 Top-3 when candidates are restricted to the five most frequent codes, and we report both because reason-code accuracy depends heavily on the label space. Because Stage 2 scores only lines Stage 1 has flagged, end-to-end performance is the product of the two stages: the correct code appears among the first three candidates for 38.8% of all denied lines. Class imbalance was addressed with the Synthetic Minority Over-Sampling Technique applied within training folds only; an ablation shows no measurable benefit at this prevalence (difference in average precision −0.0002, 95% CI −0.0009 to +0.0006) and worse calibration, which we report rather than omit. Tuned XGBoost and LightGBM baselines on the identical partition are statistically indistinguishable from the Random Forest.

**Discussion:**

The solution provides providers with a claim validation and denial-forecasting tool that reduces the denial rate and simplifies the revenue cycle.

## 1 Introduction

The rejection of insurance claims poses an extensive issue in health care. They cause significant financial and operational costs for providers, payers, and patients, particularly as AI transforms the industry (1). Every refusal request incurs a series of expenses: administrative reprocessing costs increase by approximately 15% per case, patient care times are extended, and provider-insurer tensions rise. The majority of claim management systems rely on manual reviews or rudimentary automated analyses and are incapable of handling the intricate distributions of denials (2, 3). Consequently, the time frames for reimbursement exceed 30 days, and total expenditures increase due to the systems’ inability to anticipate and respond to evolving payer policies. Recent papers have focused on predicting claim outcomes using machine learning and data-driven approaches (4, 5). The existing solutions typically construct a simple binary classification pipeline: preparing data, training a model, and providing a yes/no answer. They fail to explain why a claim was denied and to offer practical advice. Ensemble and deep learning are promising but have issues of data imbalance; they cannot be explained, and their recommendations are not detailed (6–10). These issues can be addressed using new transformer and graph neural network methods (11, 12), but they have not yet been fully applied to predict denial.

The paper presents a comprehensive predictive framework that transcends conventional workflows. PF-CDM enhances every processing stage by using its solution, which entails: (1) embedding more advanced feature engineering on 26 claim attributes; (2) applying the Synthetic Minority Over-Sampling Technique inside the training folds only, so that every validation and held-out partition retains the natural denial prevalence; and (3) an interpretable rule-based scrubber that identifies the potential reasons of denial. The framework is developed and evaluated retrospectively on professional claim lines obtained from a single U.S. healthcare payer. No prospective deployment, multi-payer trial or external validation is claimed, and the transferability of the reported results to other payers, claim types or geographies is therefore untested; this is examined in Section 6.

Figure 1 illustrates the two-stage pipeline for claim denial prediction and reason-code identification.

**Figure 1:**
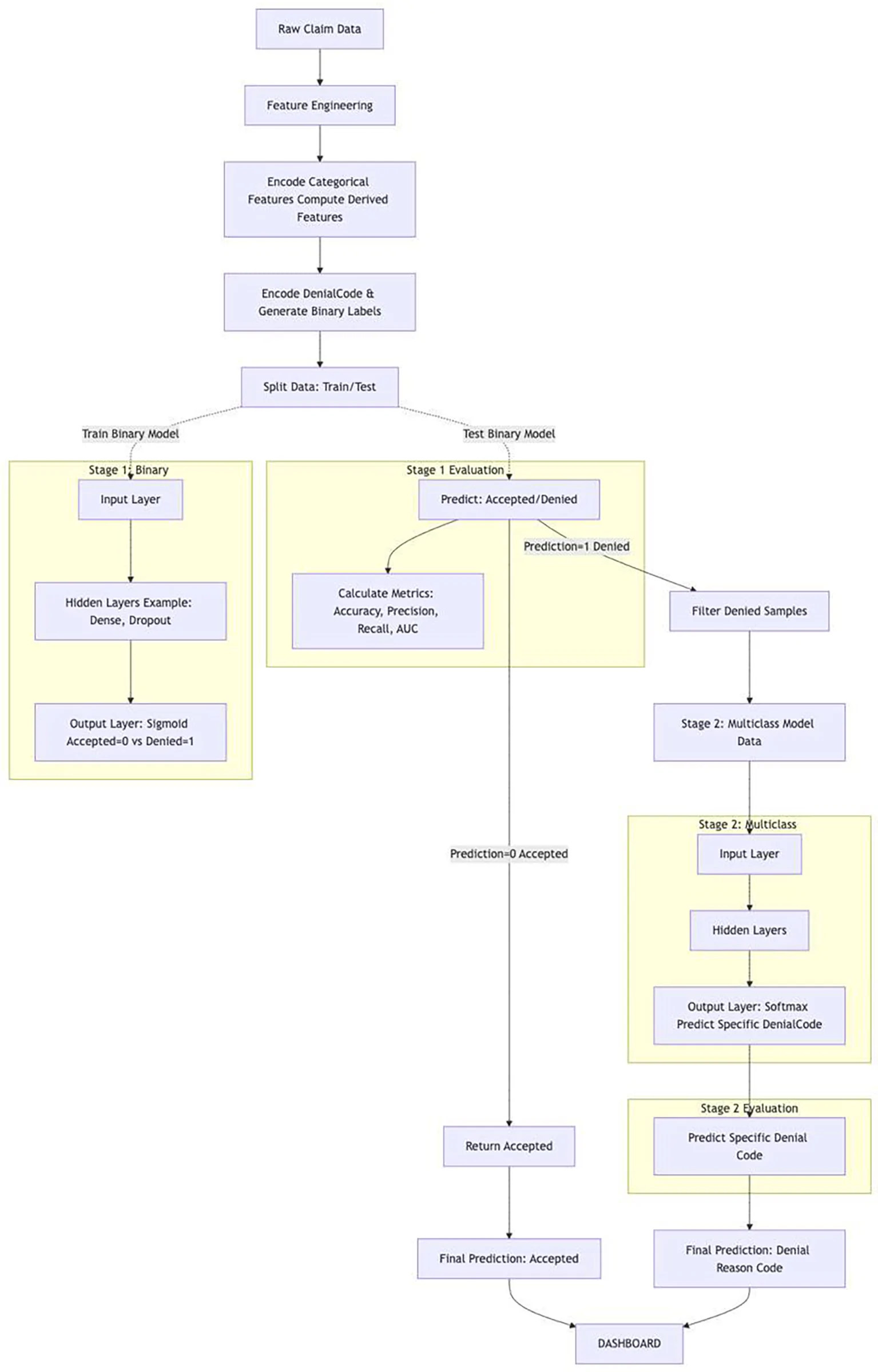
Two-stage claim denial prediction pipeline.

The significant contributions of this work are:

- A new two-step machine learning model both estimates the likelihood of a claim being denied and the code of the reason for the denial.
- A systematic feature-engineering procedure that derives 26 model-ready features from 32 raw claim fields across temporal, clinical, provider, financial and demographic dimensions. The complete feature dictionary, giving the data type, encoding scheme and point of availability of every feature, is reported in Supplementary Table S1.
- Embedding of machine learning with business rules and explainability methods, which make the system prepared to be implemented in the real world as a decision-support device.
- A quantitative leakage assessment, an ablation of the imbalance treatment, internal baselines with paired significance testing, and an end-to-end cascade evaluation, all reported with confidence intervals.

The remainder of this paper is organised as follows. Section 2 reviews prior work on claim outcome prediction, class-imbalance handling and model interpretability, and introduces the feature set. Section 3 positions PF-CDM relative to existing approaches. Section 4 describes the data source, the preprocessing and feature-engineering pipeline, the imbalance-handling strategy, the two-stage architecture, the rule-based scrubber and the evaluation protocol, including the measures taken to prevent information leakage. Section 5 reports the experimental results for both stages together with the ablation, error and feature-importance analyses. Section 6 discusses the findings, states the limitations of the study and outlines directions for future work.

## 2 Literature review

Predicting denials of healthcare insurance claims has been a long-standing research topic. Initially based on statistical techniques and primitive machine learning, the field advanced with increased computing power to employ sophisticated methods such as ensemble learning and deep learning to enhance accuracy and interpretability. Ensemble methods have consistently outperformed single learners on structured administrative and clinical data and remain the reference approach for tabular prediction tasks of this kind (7). Gradient-boosted tree ensembles in particular deliver strong accuracy at the cost of extensive hyper-parameter tuning (5), whereas the deep architectures developed for electronic health records (4) carry computational and deployment overheads that have limited their adoption in operational revenue-cycle settings (10). Coding quality is a further determinant of the denial outcome: mismatches between the submitted diagnosis and procedure codes are a recognised driver of avoidable rejection and of resubmission delay, which motivates the procedure and diagnosis category features retained by the selection procedure. A further challenge is working with lopsided data, which researchers address through techniques like SMOTE for oversampling infrequent cases (6), or by using stratified ensemble models (7). Alternative approaches include cost-sensitive learning (13) and modifications to neural network architecture (14), which form the basis for handling the inherent imbalance in claim datasets.

Model interpretability is crucial in real-world applications, especially for high-stakes decisions where transparent models are necessary (8). Methods like SHAP values (9), LIME (15), and counterfactual explanations (16) instill user confidence in the forecasts by explaining feature importance. Despite these advancements, several gaps remain. Current models primarily offer binary classification, infrequently explain the rationale for a decision, and rarely integrate domain-specific business rules; few have been successfully implemented in real business processes. As machine learning grows in importance for predicting healthcare outcomes (17) and the need to train clinicians in AI principles is recognized (18), PF-CDM aims to address these shortcomings by providing accurate predictions, precise explanations, and practical applicability.

The 26 features divide into nine temporal, six clinical, four demographic, four provider and three financial variables. Selection used permutation importance rather than impurity importance, because the latter favours high-cardinality columns such as the procedure code and the provider identifier; the two rankings differ materially and the distinction is noted in Section 5. Importance was computed over ten permutations on a random sample of 40,000 training lines, which is sufficient to separate the retained features from the discarded ones at the observed effect sizes, and the 26 features reported were those whose mean importance exceeded zero by more than one standard deviation. Every feature is computable at submission, which is a requirement for a pre-submission tool rather than an incidental property.

## 3 Comparison with existing work

Though the volume of research on predicting healthcare claim denials is increasing, existing models have limitations: they are poorly designed and have limited scope, and are not ready for use. PF-CDM framework is compared with key previous studies along four dimensions: binary accuracy, denial-code prediction, interpretability, and real-time usability. Most claim-analytics studies frame the problem as binary classification alone: the model indicates whether a claim is likely to be rejected but not why, so its output cannot be acted upon before submission. Gradient-boosted tree methods such as XGBoost (5) achieve strong general classification performance on tabular data but embed no healthcare-specific validation logic and provide no native reason attribution. Claims-specific neural approaches address adjacent problems rather than denial reason coding: the transformer-based Medicare claim encoder of Lahlou et al. (11) targets explainable health-risk estimation, the graph neural network of Hasan (12) targets fraud detection, and the hybrid ensemble of Jabeen et al. (28) forecasts insurance outcomes without producing an actionable reason code.

There is a substantive difference in the PF-CDM approach. The first stage performs binary accepted/denied classification on a held-out partition that preserves the natural denial prevalence; the complete metric suite, with 95% bootstrap confidence intervals, is reported in Table 1, and the comparison against internal baselines trained on the identical split is reported in Table 3 with the associated significance tests. The second stage estimates denial reason codes over a 67-class label space, which is a materially harder task than the five-code formulation used in much of the prior literature and is stated as such. PF-CDM further combines a pre-submission validation layer, documented with its implementation status in Appendix A, with a SHAP-based explainability layer and a dashboard that presents predicted probabilities and candidate reason codes at the point of claim entry. Such an integration makes the PF-CDM model more than a mere classifier and a decision-support system that suits clinical provider operational needs.

**Table 1:** Stage 1 performance on the held-out partition (139,615 lines, denial prevalence 33.0%, operating threshold 0.469).

Metric	Estimate	95% CI	Reference value
PR-AUC (primary)	0.7770	0.7700–0.7835	No skill 0.3302
ROC-AUC	0.8648	0.8616–0.8678	Chance 0.5000
Accuracy	0.7936	0.7905–0.7966	Majority class 0.6698
Balanced accuracy	0.7840	0.7805–0.7876	Chance 0.5000
Precision	0.6648	0.6583–0.6712	Prevalence 0.3302
Recall (sensitivity)	0.7560	0.7497–0.7626	
Specificity	0.8121	0.8084–0.8156	
$F_{1}$	0.7074	0.7021–0.7131	
Matthews correlation	0.5517	0.5444–0.5588	
Brier score	0.1429	0.1416–0.1442	Lower is better

Intervals are percentile bootstrap over 1,000 replicates resampled by claim rather than by line.

PF-CDM combines denial prediction with reason coding, model transparency, and readiness to deploy. By reporting likely denial, denial reasons, and real-time feedback and actionable insights, PF-CDM fills a research gap. The PF-CDM framework emphasises explainability, alignment with regulations, and operational feasibility, rather than the previous models that emphasised only accuracy. Explainability, as Rudin (8) argued, is non-negotiable in high-stakes areas of healthcare. This philosophy is reflected in PF-CDM, which is both statistically rigorous and clear and rule-based.

## 4 Materials and methods

### 4.1 Data source and scope

The data were obtained from a single U.S. healthcare insurance payer and comprise de-identified professional (CMS-1500) claim service lines adjudicated between October 2017 and July 2024. The unit of analysis is the service *line*, not the claim: a claim carries several lines, each with its own procedure code, service date, charge and adjudication outcome, and lines of one claim are adjudicated together. The extract contained 1,048,575 lines before cleaning and 678,881 after, distributed across 123 rendering providers and dominated by two states, Illinois (26.7% of lines) and Michigan (16.4%), with the patient state field missing on 56.3%. This is therefore a concentrated provider group in a limited geography rather than a national sample, and the generalisability implications are discussed in Section 6.

The raw extract carries 96 fields and no outcome column, so the definition of a denial is a modelling decision rather than a given. A line is treated as denied when it carries a claim adjustment reason code. The alternative rule, a non-zero denial amount, agrees with this definition on 90.8% of lines; the 9.2% disagreement consists entirely of lines carrying a reason code with no dollar amount recouped, which are denials in the operational sense and are counted as such. The resulting denial rate is 31.9% at line level. Several denominators were examined, including the dollar-weighted convention used in revenue-cycle practice, which gives 42.5%; the line-level rate is reported throughout because it is the unit the model operates on.

### 4.2 Preprocessing and exclusions

Lines missing an essential field were excluded (21,224 lines, 2.0%, all missing a procedure code), as were lines whose service date preceded the patient’s date of birth (305 lines, 0.03%). Procedure codes were normalised to five characters, ICD-10 codes were validated against the CMS pattern, payer names were standardised, and lines without a referring provider identifier were flagged rather than dropped. Dates of birth recorded with two-digit years were corrected where the parsed value fell after the claim date, a century error affecting 41,046 lines.

A further 348,167 lines, 33.9% of the extract, were exact duplicates across all fields. The extract carries no usable line identifier, so these are indistinguishable from one another and were collapsed to one instance each. This step matters beyond data hygiene: retaining duplicates and then partitioning at random places copies of the same line on both sides of the split, and a model with unconstrained leaf size then recognises rather than predicts. That mechanism is examined quantitatively in Section 5.

### 4.3 Notation

Let $D=\{(x_{i},y_{i},c_{i}){\}}_{i=1}^{N}$ denote the processed dataset, where $x_{i}\in R^{d}$ with d=26 is the feature vector of line i, $y_{i}\in \{0,1\}$ is the adjudication outcome with $y_{i}=1$ denoting denial, and $c_{i}\in C$ is the denial reason code, defined only when $y_{i}=1$. Here N=678,881 and |C|=67. Write $p=|\{i\;:\;y_{i}=1\}|/N=0.319$ for the denial prevalence.

### 4.4 Data partitioning and prevention of information leakage

The data are partitioned temporally rather than at random, and grouped on the claim. All lines belonging to claims created before 21 July 2023 form the development set $D_{\mathrm{train}}$ (539,266 lines, prevalence 0.316) and the remainder form the held-out set $D_{\mathrm{test}}$ (139,615 lines, prevalence 0.330). Grouping is necessary because lines of one claim are adjudicated together and are not independent; allowing them to straddle the partition would let the model observe part of a claim in training and the remainder in testing. Model selection uses stratified group cross-validation with five folds inside $D_{\mathrm{train}}$ only.

Four measures protect against information leakage.

First, the class prevalence of every validation fold and of the held-out partition is left untouched. Resampling is applied exclusively to the training portion of each fold, so no synthetic record is ever scored.

Second, every fitted transformation, including categorical encodings, imputation and the smoothed rate features defined in Equations 3, 4, is estimated on the training portion of the fold and then applied to the held-out portion, never the reverse. Feature selection itself is performed on the training partition alone for the same reason.

Third, fields populated only at or after adjudication are removed from the feature space before any model is fitted. This includes the denial amount, which in this extract is populated if and only if the line was denied and would therefore reproduce the target almost exactly.

Fourth, all historical and aggregate features are computed with a strict temporal cut-off placed before the index line. Because the extract carries no adjudication date, a claim submitted shortly before the index line has an outcome recorded in the data that was not knowable to the biller at submission time. The cut-off is therefore placed a fixed interval before the index claim’s creation date, and the interval was chosen empirically rather than assumed. Supplementary Table S3 reports the correlation between the history features and the outcome as the cut-off is moved back. The provider curve stabilises at 90 days, and the residual association of approximately 0.10 is the genuine provider effect; the 0.32 observed at zero lag was contamination. The patient curve is still declining at 365 days, so 365 days is used as a floor and the residual patient association is reported as an upper bound rather than an estimate.

A shuffled-label control confirms that no feature encodes the outcome of its own line. A model fitted on randomly permuted training labels and evaluated on the untouched held-out partition attained a mean ROC-AUC of 0.4835 over five permutations, with individual runs spanning 0.4231 to 0.5196, straddling chance. The control was run without the sampler, because resampling alters the geometry the test measures: with SMOTE in the pipeline the same control reads 0.5456, and a factorial check using a pure-noise feature confirmed that the elevation is an artefact of interpolation into dense regions rather than evidence of leakage.

The patient identifier merits a caveat. The extract carries no patient key, so a quasi-identifier was constructed from date of birth, sex and state, yielding 32,446 distinct values. Patients sharing all three attributes collide, and the construct is described as a proxy rather than an identifier. At the 365-day cut-off, lines from patients with three or more prior denials were denied 33.5% of the time against 32.2% for patients with a clean history, a ratio of 1.04. At a 30-day cut-off the same comparison gives 45.5% against 22.8%, which illustrates how much of an apparent patient-history effect is attributable to the cut-off rather than to patient behaviour. No patient-history feature was retained by the selection procedure.

### 4.5 Class imbalance

Stage 1 addresses the 68.1/31.9 imbalance with SMOTE (6). For a minority sample $x_{i}$, one of its k=5 minority-class nearest neighbours $x_{i}^{\left(\mathrm{nn}\right)}$ under Euclidean distance is chosen at random and a synthetic sample is generated as

$$x_{\mathrm{new}}=x_{i}+\lambda \left(x_{i}^{\left(\mathrm{nn}\right)}-x_{i}\right),\;\lambda ∼U(0,1).$$ (1)

Synthesis continues until the training-fold minority proportion reaches a sampling ratio ρ, which is tuned jointly with the forest parameters and was selected at ρ=0.7. Equation 1 is applied to training folds only. Section 5 reports an ablation showing that this treatment confers no measurable benefit at the observed prevalence.

Stage 2 does not resample. Interpolating between reason codes that are clinically distinct produces synthetic points with no meaning, so the contribution of class c to the Gini impurity at each split is weighted instead by

$$w_{c}=\frac{N_{d}}{|C|\cdot n_{c}},$$ (2)

where $n_{c}$ is the number of training lines carrying code c and $N_{d}=\sum_{c\in C}n_{c}$. Equation 2 is the inverse-frequency scheme of (7). A Random Forest is not fitted by gradient descent and has no differentiable loss, so the weighting acts on the impurity criterion at each split rather than on an objective function.

### 4.6 Derived features

The specialty risk score for taxonomy s is the empirical denial rate of that specialty, smoothed towards the global rate to stabilise sparse specialties:

$$r_{s}=\frac{\sum_{i\in T_{s}}y_{i}+\alpha \bar{\;p}}{|T_{s}|+\alpha },$$ (3)

where $T_{s}$ is the set of training-fold lines of specialty s, $\bar{\;p}$ is the training-fold denial rate and α=50 is the smoothing constant. The charge-deviation feature compares the billed amount with the payer benchmark for the same procedure and payer type:

$$\gamma_{i}=\frac{{\mathrm{Charges}}_{i}}{\mathrm{median}\{{\mathrm{Charges}}_{j}\;:\;{\mathrm{ProcCode}}_{j}={\mathrm{ProcCode}}_{i},\;{\mathrm{PayerType}}_{j}={\mathrm{PayerType}}_{i}\}}.$$ (4)

Both quantities are estimated within the training fold only.

### 4.7 Pre-submission validation layer

The deployed system applies a validation layer before a claim reaches either model. Its purpose is to reject submissions that cannot be adjudicated and to normalise those that can, rather than to anticipate a specific reason code. It is described here as implemented, and Appendix A distinguishes the capabilities that are deployed from those that are specified but not yet built.

For a single claim entered through the interface, five field-level checks must pass: a procedure code must be present, the billed charge must exceed zero, the number of units must be at least one, a drug code must be present, and the service date must precede the claim creation date, with calendar validity enforced. A submission failing any check is returned to the user rather than scored.

For a file of claims, the layer verifies that all 19 required columns are present and rejects the file otherwise. It then coerces the numeric fields, replaces values that fail coercion with zero, maps categorical values unseen during training to a baseline class so that an unfamiliar procedure code degrades gracefully rather than raising an error, and expands the payer type into four binary indicators.

Two of the manual-entry checks would reject valid claims as currently specified and are under revision; both are identified in Appendix A.

The deployed application serves the model reported here. Its interface accepts a complete claim rather than a single line, which is necessary because four of the 26 features are claim-level aggregates and two are provider-history counts; neither can be derived from one line in isolation. Provider history is computed from the submitting practice’s prior claims under the same 90-day cut-off used in training, so a line receives the same feature at inference that it would have received in training.

### 4.8 Two-stage architecture

Stage 1 is a Random Forest (19) binary classifier over x. Stage 2 is a separate multiclass Random Forest, trained on denied lines only, that estimates P(c∣x,y=1) over C. It is a single-label multiclass model, evaluated by Top-1 and Top-3 accuracy over the full label space. Because Stage 2 scores only lines that Stage 1 has flagged, a denial missed by Stage 1 is unrecoverable, and the end-to-end accuracy of the cascade is the product

$$A_{k}^{e2e}={\mathrm{Recall}}_{\mathrm{Stage}\;1}\times \mathrm{Top}\text{-}k_{\mathrm{Stage}\;2},$$ (5)

which is necessarily lower than either stage in isolation and is reported alongside the per-stage figures in Section 5.

### 4.9 Evaluation protocol

Stage 1 is assessed by accuracy, balanced accuracy, precision, recall, specificity, $F_{1}$, the Matthews correlation coefficient, ROC-AUC, the area under the precision-recall curve and the Brier score. Because the positive class is the minority, PR-AUC is treated as the primary discrimination measure and ROC-AUC as secondary. The Matthews coefficient is given by Equation 6

$$\mathrm{MCC}=\frac{TP\cdot TN-FP\cdot FN}{\sqrt{\left(TP+FP)(TP+FN)(TN+FP)(TN+FN\right)}}.$$ (6)

Calibration is assessed by the Brier score and by a reliability diagram over ten probability deciles. Stage 2 is assessed by Top-k accuracy given by Equation 7,

$$\mathrm{Top}\text{-}k=\frac{1}{\left|D_{\mathrm{test}}^{d}\right|}\sum_{i\in D_{\mathrm{test}}^{d}}1\left[c_{i}\in T_{k}(x_{i})\right],$$ (7)

where $D_{\mathrm{test}}^{d}$ is the set of truly denied held-out lines and $T_{k}(x_{i})$ the k highest-probability codes, together with macro-averaged $F_{1}$ over C.

All 95% confidence intervals are percentile bootstrap (20) over 1,000 replicates. Resampling is performed by *claim* rather than by line, because lines of one claim are not independent and a line-level bootstrap would understate every interval. Paired comparisons between classifiers on the same held-out partition use McNemar’s test for classification agreement (21) and DeLong’s test for correlated ROC curves (22), with Holm correction across the family of comparisons (23), and a bootstrap interval on the difference in average precision (20).

### 4.10 Implementation and hyper-parameters

All experiments were run in Python 3.11 with scikit-learn 1.6.1, imbalanced-learn 0.13, XGBoost, LightGBM, CatBoost and SHAP, on a four-core CPU instance with 30 GB of memory. Hyper-parameters were selected by successive-halving randomised search over a grouped 30% subsample of training claims, scored by average precision under five-fold stratified group cross-validation, and the selected configuration was refitted on the full training partition. The search space and the selected values are given in Supplementary Table S4. Random seeds were fixed for the split, the resampling and the forests. The hyper-parameter search required 41.7 min and the final refit 7.3 min on this hardware; inference over the 139,615 held-out lines completes in under a minute, which is the figure relevant to real-time use.

### 4.11 Stage 2 configuration

Stage 2 was tuned by randomised search over 20 candidates on a grouped 40% subsample of denied training claims, scored by macro $F_{1}$ under three-fold stratified group cross-validation, and refitted on all 170,636 denied training lines. The selected configuration was 500 trees, minimum leaf size 2, max_features 0.3, unrestricted depth and inverse-frequency class weighting. Codes with fewer than 100 training instances were pooled into a single residual class, leaving 67 classes covering 98.8% of denials.

### 4.12 Temporal and expert validation

Robustness to policy drift was assessed on cumulative windows of the held-out period and is reported in Section 5. Face validity of the Stage 2 output was checked informally during development, with insurance coding professionals reviewing model predictions on sampled denied lines. This was not conducted as a structured annotation study: reviewer assignments and per-line judgements were not retained, so no chance-corrected agreement statistic can be computed from it. No numeric agreement figure is therefore reported. Raw percentage agreement is in any case uninformative when a single code accounts for a large share of denials, since two raters who both default to that code will agree often while demonstrating nothing. A structured inter-rater study with stratified sampling and Cohen’s and Fleiss’ κ is required to substantiate a claim of this kind, and is identified as necessary further work in Section 6.

## 5 Experimental results

### 5.1 Stage 1: denial prediction

Stage 1 separates accepted from denied lines on the held-out partition, which retains the natural denial prevalence of 33.0%. At the operating threshold of 0.469, selected by maximising $F_{1}$ on the validation folds, the confusion matrix in Figure 2 records 75,941 correctly accepted lines, 34,850 correctly identified denials, 17,574 lines incorrectly flagged and 11,250 denials missed. Every quantity in Table 1 follows from those four counts or from the predicted probabilities, so the table and the figure are mutually reconcilable: accuracy is (75,941+34,850)/139,615=0.794.

**Figure 2:**
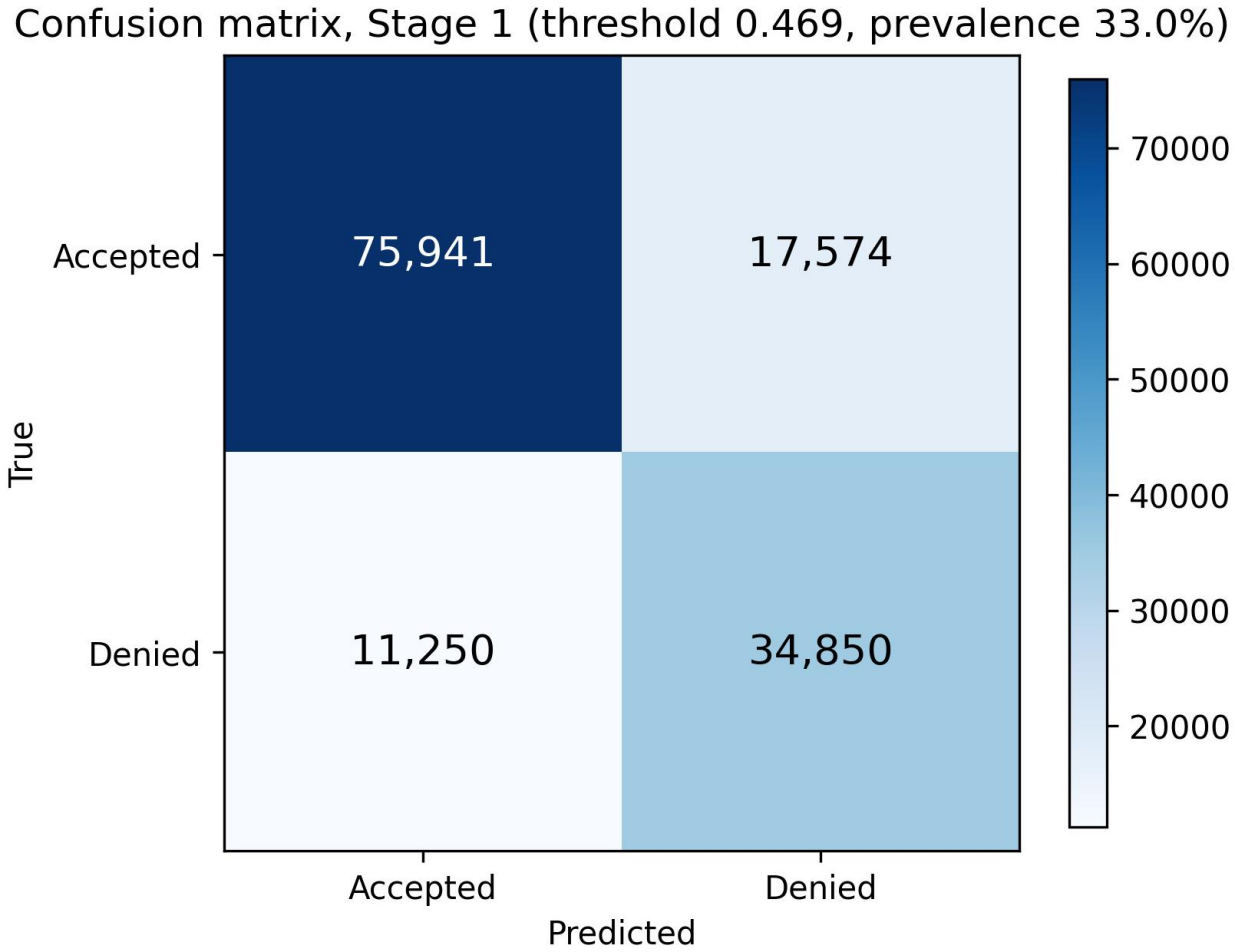
Binary confusion matrix at the stated threshold, drawn at the natural denial prevalence of 33.0%.

PR-AUC is the primary measure because the positive class is the minority and accuracy is prevalence-dependent: predicting “accepted” for every line scores 0.670 here. The model’s 0.794 is a gain of 12.4 points over that floor, and its PR-AUC of 0.777 is 2.35 times the no-skill baseline.

Figure 3 presents the ROC and precision–recall curves for Stage 1.

**Figure 3:**
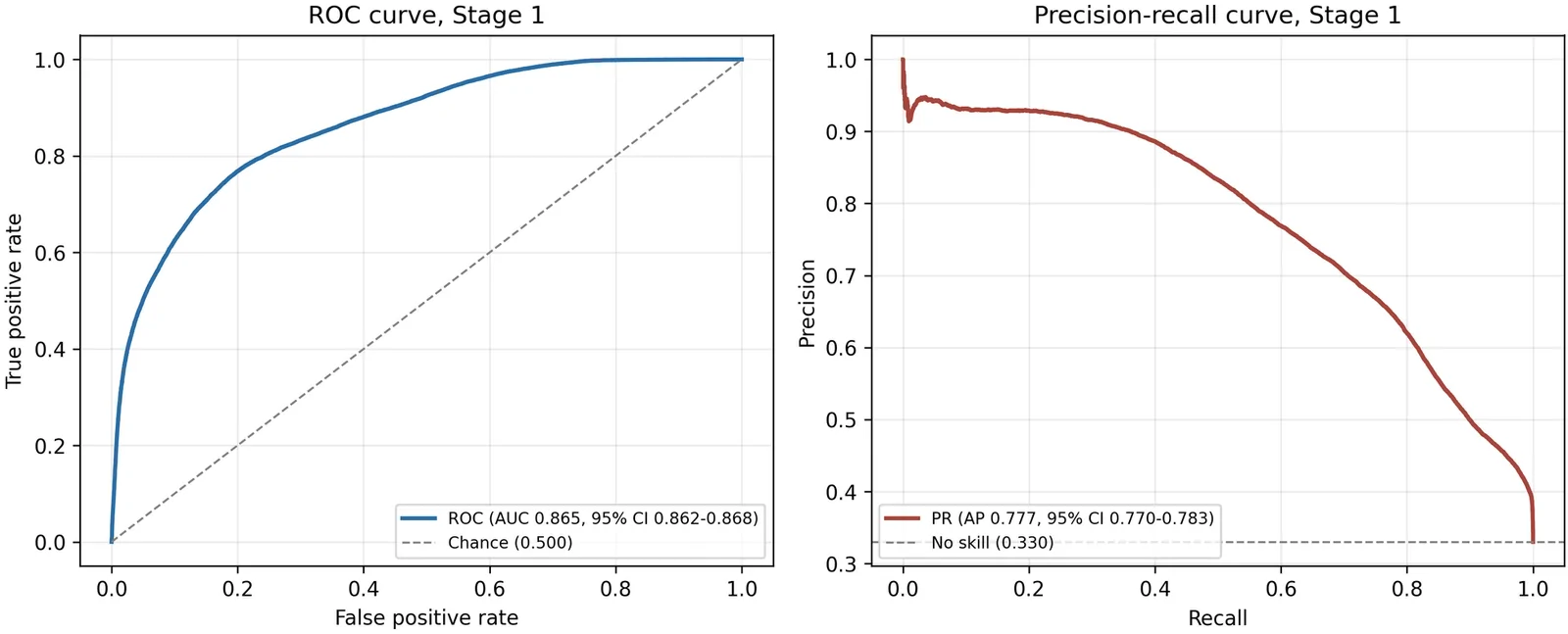
ROC and precision-recall curves for Stage 1, with bootstrap intervals in the legend and the no-skill line drawn at the prevalence.

### 5.2 Calibration and operating points

A tool that presents a biller with a denial probability must be calibrated, not only discriminative. The Brier score is 0.143 and the expected calibration error over ten quantile bins is 0.071, adequate for triage but not for a probability quoted to a payer. Figure 4 shows the calibration curve and predicted probability distribution for Stage 1. Table 2 restates the model in the terms a revenue-cycle team works in.

**Figure 4:**
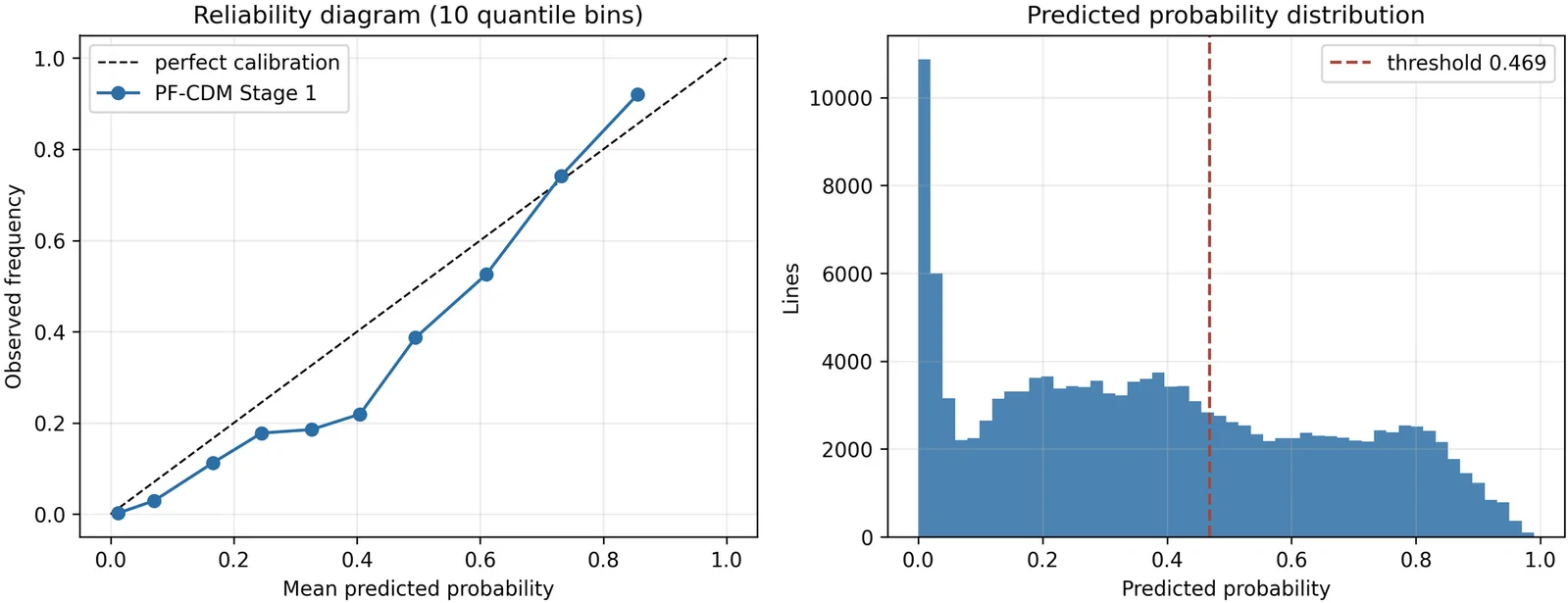
Reliability diagram over ten quantile bins, with the distribution of predicted probabilities and the operating threshold.

**Table 2:** Stage 1 operating points.

Threshold	Lines flagged	Precision	Recall	False alarms per denial caught
0.367	50%	0.559	0.846	0.79
0.446	40%	0.644	0.780	0.55
0.549	30%	0.729	0.663	0.37
0.671	20%	0.831	0.503	0.20
0.790	10%	0.920	0.279	0.09
0.847	5%	0.930	0.141	0.08

Each row flags a fixed share of submitted lines for pre-submission review.

### 5.3 Comparison with internal baselines

Six comparators were tuned on the identical grouped subsample with the same number of search draws and refitted on the full training partition. Table 3 reports performance and Table 4 the paired tests. The Random Forest has the highest PR-AUC, but its margin over tuned XGBoost and LightGBM is not significant: the bootstrap intervals on the difference in average precision span zero and DeLong’s test does not reject. It is significantly ahead of CatBoost, a single decision tree and logistic regression. Figure 5 compares PF-CDM with the baseline models using PR-AUC.

**Figure 5:**
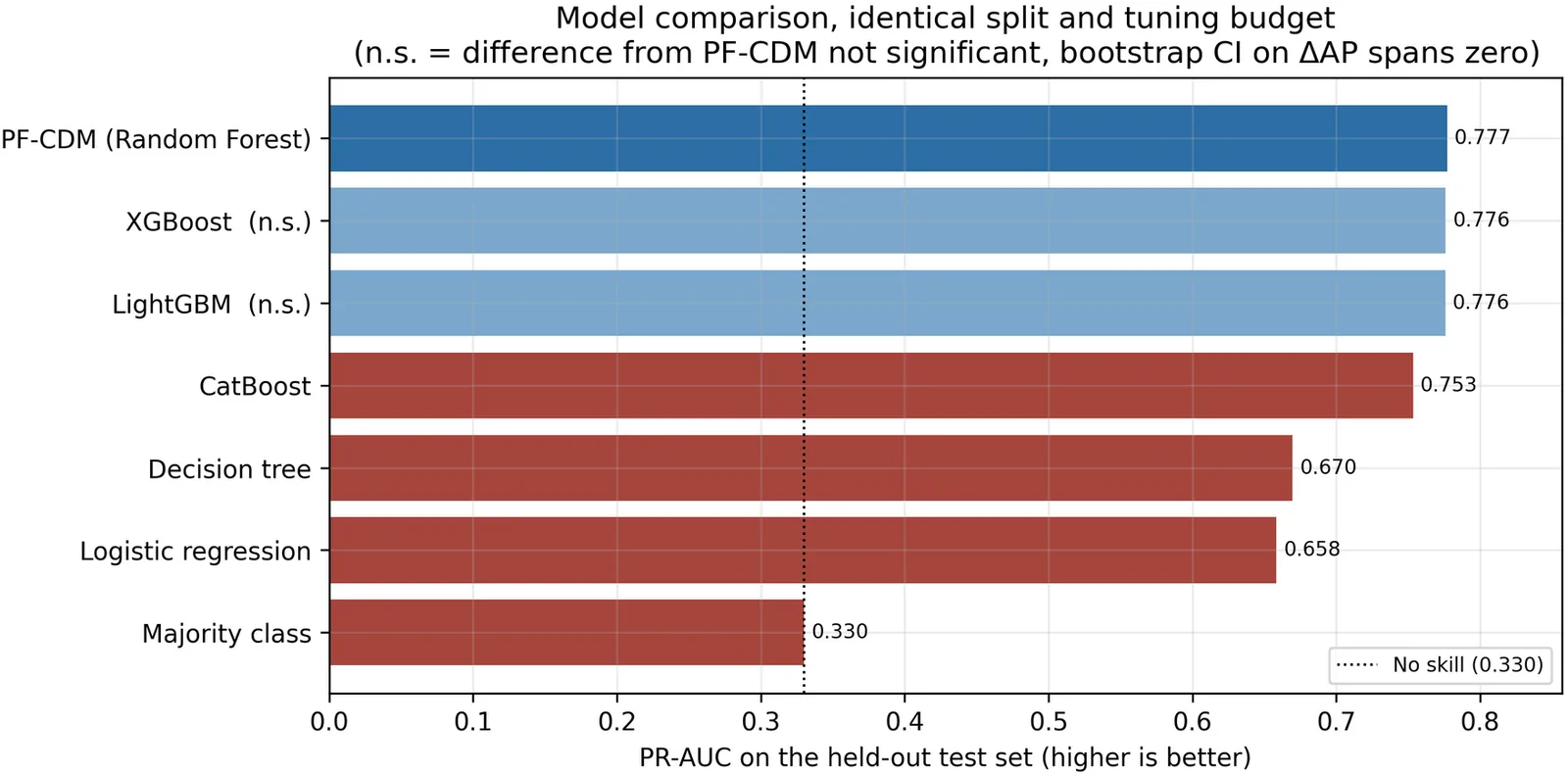
Model comparison on PR-AUC, identical split and tuning budget, with the no-skill line drawn. Comparators whose difference from PF-CDM is not statistically significant are marked. The metric is stated on the axis and the no-skill line is drawn, so the bars cannot be read as accuracy.

**Table 3:** Internal baselines on the identical partition, feature set and tuning budget.

Model	PR-AUC	ROC-AUC	Bal. acc.	$F_{1}$	MCC	Brier
**PF-CDM (Random Forest)**	**0.7770**	0.8648	0.7840	0.7074	0.5517	0.1429
XGBoost	0.7761	0.8643	0.7841	0.7077	0.5523	0.1453
LightGBM	0.7758	0.8639	0.7832	0.7089	0.5623	0.1426
CatBoost	0.7533	0.8652	0.7847	0.7086	0.5545	0.1431
Decision tree	0.6697	0.8223	0.7537	0.6688	0.4817	0.1812
Logistic regression	0.6585	0.8149	0.7383	0.6510	0.4515	0.2060
Majority class	0.3302	0.5000	0.5000	0.4965	0.0000	0.2214

PR-AUC is the primary metric, and every model was refitted on the full training partition at its selected configuration.

**Table 4:** Paired tests against Stage 1.

Comparison	ΔAP	95% CI	McNemar p	DeLong p	Conclusion
vs XGBoost	+0.0008	−0.0018 to +0.0034	0.415	0.634	not significant
vs LightGBM	+0.0011	−0.0016 to +0.0041	<0.001	0.267	see text
vs CatBoost	+0.0235	+0.0204 to +0.0269	0.004	0.391	PF-CDM ahead
vs Decision tree	+0.1072	+0.1037 to +0.1110	<0.001	<0.001	PF-CDM ahead
vs Logistic regression	+0.1185	+0.1136 to +0.1227	<0.001	<0.001	PF-CDM ahead

ΔAP is the difference in average precision, PF-CDM minus the comparator, with a bootstrap 95% interval; p-values are Holm-corrected across the family.

One nuance deserves comment. McNemar’s test rejects for LightGBM although the discrimination measures do not. The two models rank lines almost identically but settle at different operating points: LightGBM’s threshold yields higher precision, 0.699 against 0.665, and lower recall, 0.720 against 0.756. The disagreement is about where the threshold falls, not about the ability to separate the classes.

### 5.4 Ablation of the imbalance treatment

At a denial prevalence of 31.9% the class imbalance is mild, so the contribution of the resampling step warrants testing rather than assumption. Supplementary Table S5 reports the ablation. The difference in average precision between the resampled and unsampled variants is −0.0002 with a 95% interval of −0.0009 to +0.0006: the interval spans zero and is narrow enough to be conclusive rather than merely underpowered. The unsampled variant is better calibrated, Brier 0.140 against 0.145, and fits in roughly half the time. SMOTE is retained in the reported pipeline because the difference is not adverse and the method is widely used for this class of problem, but the evidence here is that it is unnecessary at this prevalence.

### 5.5 Feature contribution

Table 5 reports the ablation of feature categories. All variants share one estimator configuration and no resampling, so the differences are like for like; the reference row is that configuration with all 26 features, which reaches 0.786. Financial features carry the great majority of the signal, and withholding the four provider features changes PR-AUC by nothing at all. Claim economics, rather than provider identity, carry the predictive signal in these data.

**Table 5:** Feature-category ablation.

Category withheld	Features left	PR-AUC	Change	Share of total loss
None (all 26 features)	26	0.7859	–	–
Financial	23	0.7245	−0.0614	61.5%
Clinical	20	0.7641	−0.0218	21.8%
Temporal	17	0.7772	−0.0087	8.7%
Demographic	22	0.7780	−0.0079	7.9%
Provider	22	0.7859	−0.0000	0.0%

The reference row uses the same estimator and no resampling as the variants, which is why it differs from Table 1. Shares are computed from the measured loss when each category is withheld.

**Table 6:** The five most frequent denial reason codes in the training partition, with both denominators reported.

Code	Description	Lines	% of denials	% of all lines
16	Claim lacks information needed for adjudication	20,074	11.76	3.72
B9	Patient is enrolled in a hospice	13,562	7.95	2.52
97	Benefit included in payment for another service	12,265	7.19	2.27
109	Not covered by this payer; submit to the correct one	11,196	6.56	2.08
96	Non-covered charge	7,310	4.28	1.36

Code B9, hospice enrolment, is the second most frequent and is an eligibility condition answerable before submission.

**Table 7:** Stage 2 comparators on the held-out denied lines, over the full 67-class label space.

Model	Top-1	Top-3	Top-5	Macro $F_{1}$
PF-CDM (Random Forest)	0.2685	0.4975	0.6176	0.2206
Decision tree	0.1425	0.2375	0.2980	0.1208
Majority class (code 16)	0.1416	–	–	0.0000

The majority-class row predicts the most frequent code for every line and is the floor any useful model must clear.

**Table 8:** End-to-end cascade performance at the Stage 1 operating threshold.

Evaluation Scope	Top-1	Top-3	Top-5
Stage 2 alone, all denied lines	0.269	0.498	0.618
Stage 2 conditional on Stage 1 flagging	0.287	0.514	0.630
End to end (Stage 1 recall 0.756)	0.217	0.388	0.476

Conditional accuracy is measured on the denied lines Stage 1 flagged; the end-to-end figure is the product with Stage 1 recall and is necessarily lower than either stage alone.

The SHAP analysis agrees: the strongest contributions come from the total charge of the claim, the submission lag, the line’s share of the claim total and the number of lines on the claim. Exact TreeSHAP (24) is intractable for the deployed forest, whose unconstrained leaves number in the hundreds of millions, so attributions were computed on a constrained surrogate fitted on the same data and features, over a random sample of 3,000 held-out lines. Surrogate fidelity is measured rather than assumed: PR-AUC 0.775 against the deployed model’s 0.777, and a Spearman rank correlation of 0.961 between the two models’ predicted probabilities on the held-out partition. Dependence plots for the two strongest contributors are given in Supplementary Figures S4, S5. This ranking differs from one based on impurity, which favours high-cardinality columns such as the procedure code and the provider identifier; permutation and SHAP attributions are reported here for that reason.

Figure 6 summarizes feature contributions using SHAP values from the surrogate model. A worked example makes the output concrete. Local explanations for all three error modes are given in Supplementary Figures S6–S8: a true positive, a false positive and a false negative drawn from the same sample. For the false positive in Supplementary Figure S7 the model returned a denial probability of 0.62 for a line that was subsequently paid. The three largest contributions were the claim total of USD 605.44, the line’s small share of that total (4.3%) and a submission lag of four days. The model had learned that low-share lines on larger claims are frequently bundled and denied under code 97; here the bundling did not apply. A biller reviewing this line would confirm the bundling status and release it, at the cost of one review. For contrast, the false negative in Supplementary Figure S8 carried a claim total of USD 200.00 with a submission lag of 276 days and received a probability of 0.28: the model weighted the small charge more heavily than the extreme lag, and the line was denied for timely filing. These are the two error modes a deployment should monitor.

**Figure 6:**
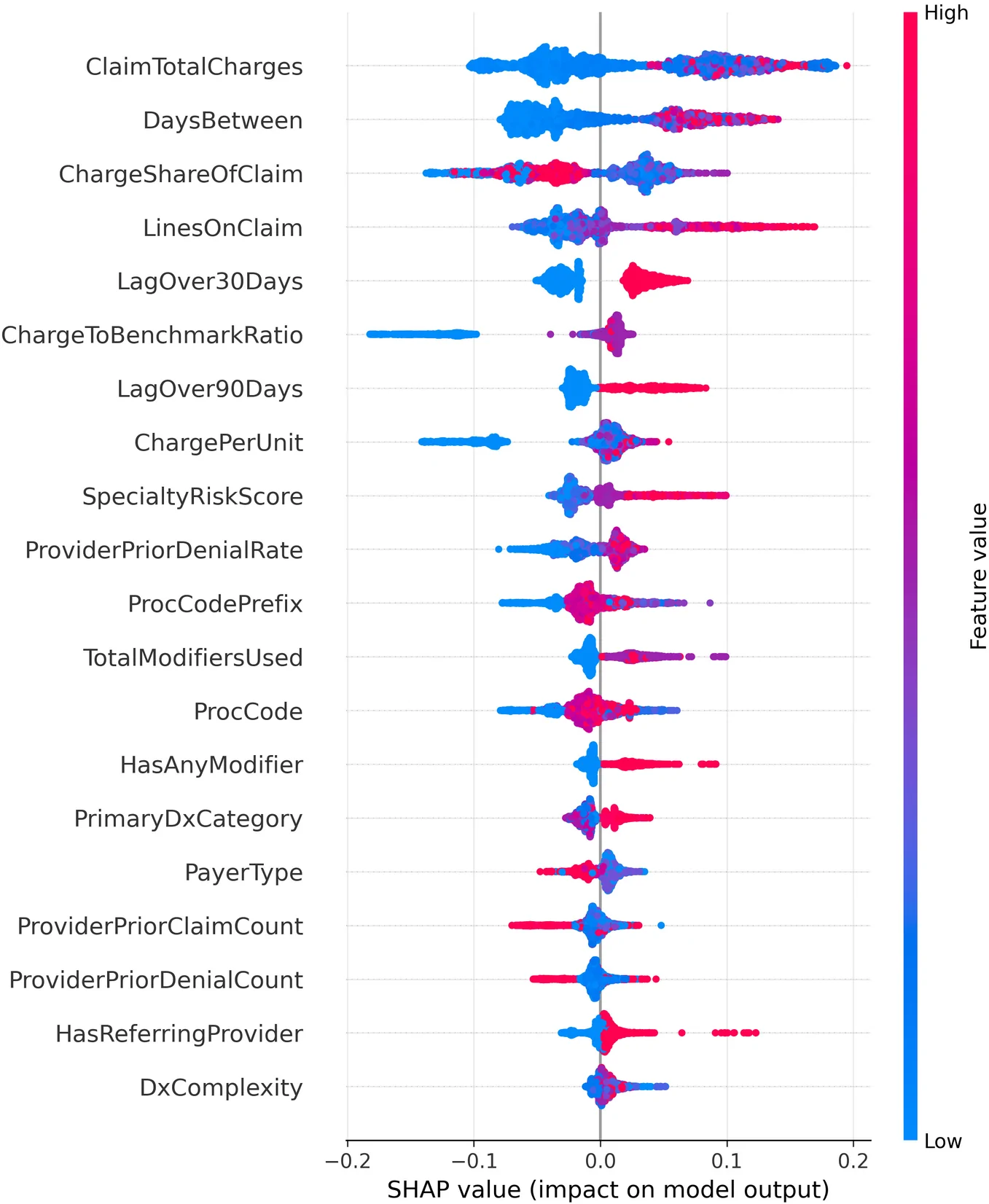
SHAP summary over held-out lines, computed on a fidelity-checked surrogate (Spearman ρ=0.961 against the deployed model).

### 5.6 Generalisation and drift

Two evaluations bound the claim of operational readiness. Withholding 24 of the 123 rendering providers entirely and training on the remainder gives a PR-AUC of 0.677 on 284,349 lines from those providers, against 0.777 on the temporal split. The temporal split answers the leakage question but not the question of generalisation to an unfamiliar practice, since 95.4% of held-out lines come from a provider present in training. We report both: the temporal figure describes the deployed setting for this provider group, the grouped figure what a new practice should expect.

Performance also declines across the held-out period, from PR-AUC 0.881 over the first month to 0.777 over twelve. Part of this is base-rate movement rather than model degradation: the denial prevalence falls from 0.448 to 0.330 over the same windows and PR-AUC depends on prevalence, whereas ROC-AUC does not and falls by only 0.035. Reporting the metric alongside the prevalence is necessary, since a decline in PR-AUC across a period of falling prevalence does not by itself indicate model degradation.

### 5.7 Subgroup performance

Performance was measured across payer type, provider specialty, patient state, age band and sex, and is reported in Supplementary Table S11 with subgroup sizes, and the parity measures on the largest contrast within each attribute in Supplementary Table S12. Discrimination varies considerably by payer, from a PR-AUC of 0.652 on Medicare lines to 0.938 on Medicaid lines, but this tracks the denial prevalence of those subgroups, 25.9% and 59.6% respectively, and PR-AUC depends on prevalence; ROC-AUC, which does not, spans the narrower range 0.793 to 0.924. Race and ethnicity are not recorded in this extract, so no analysis of racial disparity is possible; we state this rather than omit it. The consequence of an error also bears stating: a false positive causes a claim line to be held for pre-submission review, not care to be withheld, which materially changes the harm profile relative to clinical prediction settings.

### 5.8 Stage 2: denial reason coding

Stage 2 predicts the reason code for a denied line over 67 classes, comprising the 66 codes with at least 100 training instances plus a pooled residual class, together covering 98.8% of denials. On 46,100 held-out denied lines it attains 0.269 Top-1 (95% CI 0.263–0.274), 0.498 Top-3 (0.491–0.504) and 0.618 Top-5 (0.611–0.624), with macro $F_{1}$ of 0.221 and weighted $F_{1}$ of 0.264. The two $F_{1}$ figures are close, which indicates performance is not concentrated in a single frequent code.

Reason-code accuracy depends strongly on how many codes are admitted as candidates, and Supplementary Table S7 reports that dependence explicitly because it is a common source of incomparability in this literature. Restricted to the five most frequent codes the same model reaches 0.918 Top-3; restricted to three, 0.708 Top-1. Reason-code results reported in this literature are therefore not comparable unless the label space is stated, and we report the full 67-class figures as the headline result with the restricted values as a secondary analysis. Figure 7 illustrates how reason-code accuracy varies with the size of the candidate label space.

**Figure 7:**
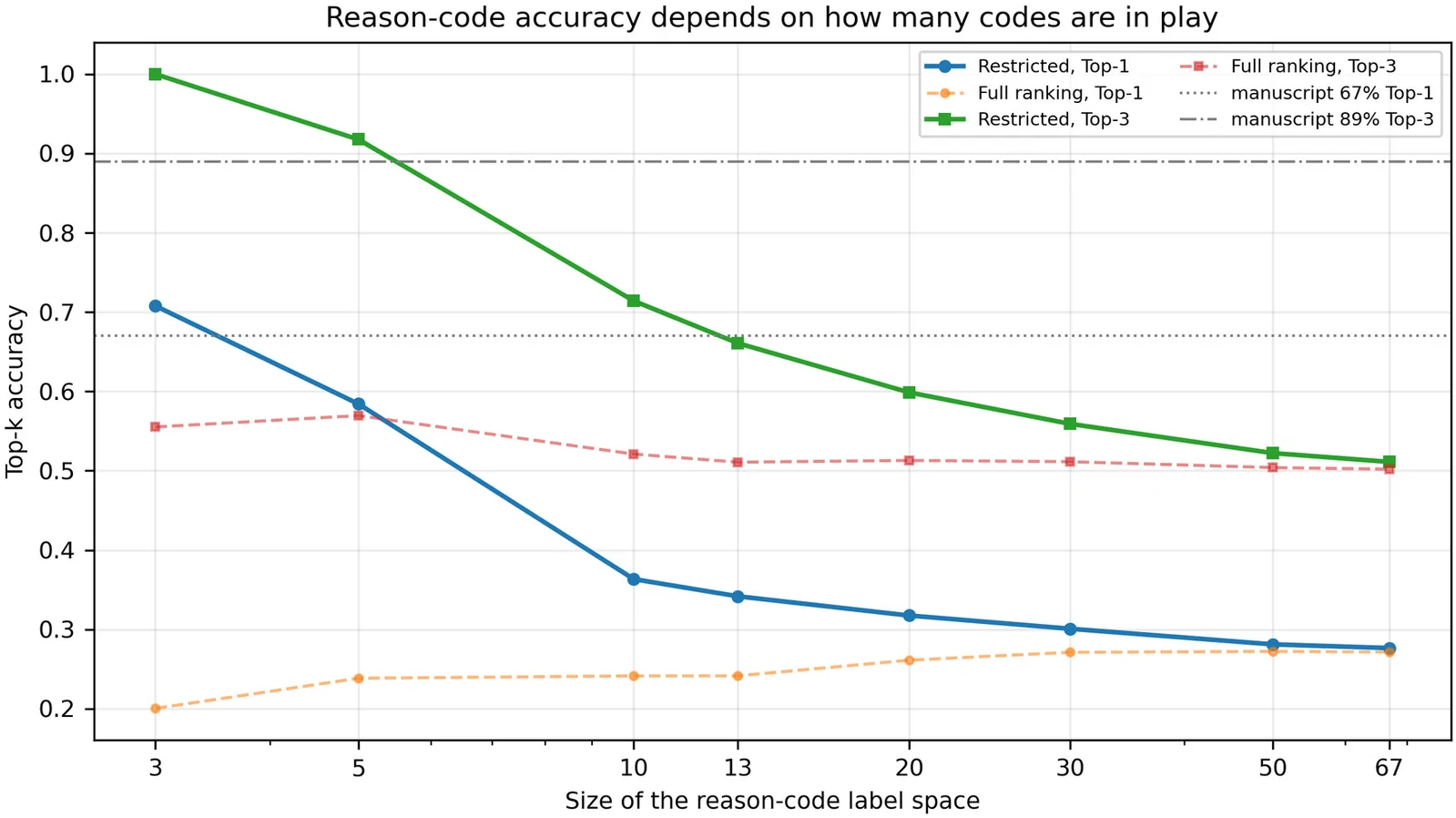
Reason-code accuracy against the size of the label space. Accuracy rises sharply as the candidate set narrows, which is why the label space must be stated alongside any reported figure.

The error structure is systematic rather than diffuse. The largest confusions are code 29 predicted as A1 (28.9% of its instances), code 96 as A1 (23.9%), code 31 as 18 (23.9%) and code 24 as 109 (23.4%). Codes 29, 96 and A1 are all coverage and eligibility denials; codes 24 and 109 both concern which payer is responsible. The model confuses categories that are adjacent in adjudication practice, which is a more useful limitation than a general statement about rare codes and indicates where further features would help.

Stage 2 was compared against three alternatives trained on the identical denied-line partition with the same feature set. The Random Forest leads by a wide margin, in contrast to the binary stage where tuned gradient boosting matched it: the 67-way task separates learners that the binary task does not.

Two further comparators were excluded rather than reported. Multinomial logistic regression reached 0.1059 Top-1 and gradient boosting 0.0361, both below the majority-class floor of 0.1416. A model that loses to predicting the single most frequent code for every line is misconfigured rather than outperformed, and we record the failures rather than present either as a result. The decision tree clears the floor by only 0.0009, which is itself an indication of how hard 67-way reason-code assignment is on these features.

### 5.9 The two-stage cascade

Stage 2 scores only lines that Stage 1 has flagged, so the operational performance of the deployed pipeline is the product of the two stages, as given in Equation 5. At the Stage 1 operating threshold, 37.6% of all submitted lines are flagged, Stage 1 recovers 75.6% of denials, and Stage 2 places the correct code among its first three candidates for 51.4% of those. End to end, the correct reason code appears among the first three candidates for 38.8% of all denied lines, and is the single top candidate for 21.7%.

We quote no single combined accuracy for the framework, because none exists independently of the Stage 1 threshold: the cascade moves with the operating point. Reporting the two stages separately without their product would overstate what a billing team experiences.

## 6 Discussion and future work

PF-CDM predicts the denial of a submitted claim line and, for lines predicted to be denied, the associated reason code, at a level that is operationally useful within the payer studied. Because the studies in Supplementary Table S2 address different tasks on different datasets at different prevalences, we make no claim of state-of-the-art performance against them. The appropriate comparators are the internal baselines of Table 3, against which the Random Forest is indistinguishable from tuned gradient boosting and clearly ahead of linear and single-tree models. The contribution of this work is therefore the two-stage architecture, the scrubber and the evaluation protocol rather than the choice of learner, and we state that plainly.

### 6.1 Practical implications for denial management

The operating-point analysis in Table 2 translates the model into the quantity a revenue-cycle team actually plans against, which is reviewer workload. Over the twelve-month held-out period the payer adjudicated 139,615 professional lines, of which 46,101 were denied. Flagging the 20% of lines with the highest predicted risk would place 27,923 lines in a pre-submission review queue and surface 23,189 of those denials, at a cost of 4,734 unnecessary reviews, or roughly one false alarm for every five denials caught. Flagging 30% raises the yield to 30,565 denials but triples the false alarms to 11,319. The choice between these is a staffing decision rather than a modelling one, and the contribution of the model is that it makes the trade-off explicit and tunable rather than fixed.

The reason-code stage changes what a reviewer does with a flagged line. Without it the queue is undifferentiated and every line requires the same manual diagnosis; with it, each flagged line arrives with three candidate reason codes, and the correct code is among them for 38.8% of denied lines end to end. For the codes the model identifies most reliably this is directly actionable: a line flagged with code 29 has a 41.6% chance of being a timely-filing denial, which is checkable in seconds against the submission date.

The error analysis also points to a preventive measure that requires no model at all. Code B9, hospice enrolment, accounts for 7.95% of denials, approximately 3,665 lines over the held-out period, and is an eligibility condition that a payer eligibility query answers before submission. The same applies to the cluster of codes 29, 96 and A1 that the reason-code model confuses with one another: these are coverage and eligibility determinations rather than coding errors, and are better addressed by an eligibility check at entry than by a more accurate classifier. A realistic deployment therefore pairs the model with the eligibility and coverage capabilities set out in Appendix A, and we regard that pairing rather than the classifier alone as the operational contribution of this work.

Two constraints bound what a provider should expect. The model is calibrated well enough for triage but not for quoting a probability to a payer, with an expected calibration error of 0.071, so its output should be presented as a ranking rather than as a percentage likelihood. And performance on a practice the model has not seen falls from 0.777 to 0.677 in PR-AUC, so a new deployment should expect a period of degraded accuracy until local claims enter the training window.

The feature analysis points to claim economics rather than provider identity: the total charge of the claim, the line’s share of that total and the charge per unit together account for the largest part of the model’s discriminative power, followed by submission timing. Provider identity and specialty contribute nothing measurable once these are present. Denial risk in this dataset is driven more by what is billed, and how a line sits within its claim, than by who submitted it.

Several limitations bound these results. First, all data originate from a single U.S. payer, so the labels encode that payer’s adjudication policy, edit configuration and reason-code conventions. The provider base is narrow: 123 rendering providers, the ten largest accounting for 73.6% of lines, operating predominantly in two states. No external dataset was available, and under a provider-held-out split PR-AUC falls to 0.677, which is the realistic expectation for an unfamiliar practice. Second, the study covers professional CMS-1500 lines only; institutional, dental and pharmacy claims follow different adjudication paths. Third, although every feature is restricted to information available at submission and the shuffled-label audit found no residual signal, retrospective data cannot exclude an unobserved upstream process that correlates a feature with the outcome.

Fourth, performance decreases on rare reason codes (6): those with fewer than 100 training instances were pooled and are not individually predictable. Fifth, the framework assigns one code per line, whereas real denials frequently carry several, which the Top-3 formulation mitigates but does not resolve. Sixth, bias is addressed only descriptively. Subgroup performance is reported across payer type, specialty, state, age band and sex, but the extract carries neither race nor ethnicity nor an income proxy, so the disparities most often examined in the fairness literature (25) cannot be measured. Two further sources deserve note: the labels encode one payer’s decisions, so any systematic bias in those decisions is reproduced by a model trained to predict them, and the concentrated provider base confounds specialty and geography with provider identity. This is the most significant gap in the present evaluation.

Seventh, no structured expert validation of the reason-code output has been carried out. Face validity was checked informally during development, but without retained per-line annotations no chance-corrected agreement can be reported, and none is claimed. Eighth, the extract as supplied was truncated at 1,048,576 rows, the maximum a spreadsheet application will hold, by an export step upstream of this study; the analysis therefore covers a contiguous slice spanning the full 2017 to 2024 period rather than the payer’s entire data. Ninth, the evaluation is retrospective throughout.

One methodological observation generalises beyond this dataset. Balancing a claims extract and then partitioning it produces optimistic results through two distinct mechanisms. A balanced held-out partition raises accuracy and precision through the base rate alone, with no change in the model. And if the balancing duplicates or synthesises rows before the split, copies of the same record appear on both sides, and a model with unconstrained leaf size recognises rather than predicts. Neither mechanism is visible in the headline metrics, which is what makes the practice hazardous. The remedy is to resample inside the cross-validation folds and to leave every held-out partition at its natural prevalence.

Four directions follow from the findings rather than from general aspiration. The first is a prospective evaluation: whether presenting these predictions to billing staff reduces the denial rate is the only question that would justify deployment, and it cannot be answered retrospectively. The second is the coverage and eligibility capability specified in Appendix A, which the error analysis identifies as the largest available gain, since the codes the model most often confuses are eligibility determinations rather than coding errors. The third is a multi-payer extract, which would separate the payer’s adjudication policy from claim characteristics that generalise. The fourth is a multi-label formulation, since real denials frequently carry several reason codes and the present single-label treatment is an approximation. Transfer learning from frequent to rare codes (26) and text features drawn from denial correspondence (27) are plausible extensions, but both are secondary to establishing that the framework changes outcomes in practice.

PF-CDM offers healthcare providers a calibrated, interpretable tool for pre-submission claim review, and an evaluation protocol that other work in this area can adopt. We regard the explicit account of what the model cannot do as part of the contribution rather than a caveat on it.

## Data Availability

The raw data supporting the conclusions of this article will be made available by the authors, without undue reservation.

## Appendix A Scrubber specification, implementation and measurement

A scrubber specification and a scrubber build are different things, and the distinction determines what a reader can rely on. This appendix separates them: the specification the system was designed against, the subset currently implemented, and a measurement of what the implemented rules achieve.

### A.1 Design specification

The scrubber was specified against published sources rather than assembled ad hoc: CMS guidance, National Correct Coding Initiative edits, and Local and National Coverage Determinations (LCDs and NCDs), together with payer-specific contract terms. The specification covers four capability groups: initial claim validation, advanced rule-based scrubbing, error detection and correction, and rule management with versioning. Supplementary Table S8 lists each specified capability with its current implementation status, so that a reader can distinguish design intent from delivered function.

Four of the thirteen specified capabilities are deployed in the application and three more are implemented in the preprocessing pipeline described in Section 4. The six remaining, which include the NCCI consistency edits and the medical-necessity and coverage checks, are designed but not built. No capability in the specification maps a claim condition to a specific denial reason code; that function is provided by Stage 2.

### A.2 Validation layer as built

Supplementary Table S9 lists the checks implemented in the released application and in the preprocessing pipeline. Six govern manual entry and batch upload, four are applied during preprocessing of the study extract, and three normalise field values before scoring. None of them anticipates a denial reason code.

Two of the manual-entry checks would reject valid claims as currently specified. Requiring a non-empty drug code excludes every line that is not a drug administration, which is the large majority. Requiring a charge strictly greater than zero excludes zero-charge quality-reporting codes such as CPT 3078F, which appear legitimately in the extract. Both are under revision, and we report them rather than describe an idealised layer.

### A.3 Candidate reason-code rules

To establish whether a reason-code rule layer is worth building, three candidate rules were specified from payer policy and national coding edits and then measured on the held-out partition. Supplementary Table S10 reports, for each, the number of held-out denied lines on which it fires and its positive predictive value (PPV), the share of those carrying the anticipated code.

Only the timeliness rule is precise enough to act on unaided, identifying its code in 41.6% of the lines on which it fires. The other two are better understood as flags for human review: the duplicate check in particular fires on many lines and identifies the associated code in fewer than one in twenty. We report this rather than present three rules as though all were validated controls.

The measurement also indicates where the specified but unbuilt capabilities would pay. Code B9, hospice enrolment, accounts for 8.0% of denials in this extract and is an eligibility condition fully answerable before submission, so the specified coverage-validation capability would prevent it outright. Codes 29, 96 and A1, which the reason-code model confuses with one another (Supplementary Figure S8), are likewise coverage and eligibility denials rather than coding errors. The two capabilities most worth building next are therefore coverage validation and the NCCI consistency edits, in that order, and this is an empirical conclusion from the error analysis rather than a restatement of the specification.
