# Supplementary material for: A machine learning framework for predicting healthcare insurance claim denials
Source: Front Health Serv. 2026 Oct 14;6:1927175. doi: 10.3389/frhs.2026.1927175 (PMC13713848; doi:10.3389/frhs.2026.1927175)
Supplement: Supplementary file 1 [file Supplementaryfile1.pdf]

Supplementary Material

1 SUPPLEMENTARY TABLES

Table S1. Feature dictionary. All 26 features were selected by Random Forest permutation importance computed on the training partition only, from a pool of 56 engineered candidates. "Available" indicates the value is computable from information carried on the claim at submission, using only records timestamped strictly earlier than the claim creation date. The reason code is the Stage 2 target and the post-adjudication denial amount is excluded from the feature space entirely.

Rank	Feature	Category	Encoding or unit	Avail.
1	ClaimTotalCharges	Financial	Sum of charges over all lines of the claim, USD	Yes
2	PayerType	Demographic	Commercial, BCBS, Medicaid or Medicare	Yes
3	DaysBetween	Temporal	Claim creation date minus service date, days	Yes
4	LinesOnClaim	Provider	Count of service lines on the claim	Yes
5	ChargeShareOfClaim	Financial	Line charge divided by the claim total	Yes
6	PatientAge	Demographic	Years at date of service	Yes
7	ProviderPriorClaimCount	Provider	Prior lines by the same NPI, 90-day cut-off	Yes
8	ProviderPriorDenialCount	Provider	Prior denials by the same NPI, 90-day cut-off	Yes
9	ClaimCreationDay	Temporal	Day of month, 1–31	Yes
10	LagOver30Days	Temporal	Indicator, submission lag over 30 days	Yes
11	HasReferringProvider	Provider	Indicator, referring provider identifier present	Yes
12	HasAnyModifier	Clinical	Indicator, at least one CPT modifier present	Yes
13	ClaimCreationDayOfWeek	Temporal	Day of week, 0–6	Yes
14	AgeBand	Demographic	0–17, 18–44, 45–64, 65–74, 75+	Yes
15	ProcCode	Clinical	CPT or HCPCS code, five characters, ordinal encoded	Yes
16	ServiceDay	Temporal	Day of month, 1–31	Yes
17	ClaimCreationMonth	Temporal	Month, 1–12	Yes
18	ServiceMonth	Temporal	Month, 1–12	Yes
19	HasSecondaryPayer	Demographic	Indicator, secondary payer present	Yes
20	TotalModifiersUsed	Clinical	Count of CPT modifiers on the line, 0–2	Yes
21	LagOver90Days	Temporal	Indicator, submission lag over 90 days	Yes
22	ServiceDayOfWeek	Temporal	Day of week, 0–6	Yes
23	PrimaryDxCategory	Clinical	First three characters of the primary ICD-10 code	Yes
24	ProcCodePrefix	Clinical	First three characters of the procedure code	Yes
25	DxComplexity	Clinical	Diagnosis count scaled by modifier count	Yes
26	ChargePerUnit	Financial	Line charge divided by units billed, USD	Yes

Table S2. Positioning of PF-CDM relative to the cited literature. Reported accuracies are omitted because the cited works evaluate different tasks on different datasets at different class prevalences, and figures obtained under those conditions are not comparable. Quantitative comparison is instead made against internal baselines trained on the same partition, in Table 3 of the main text. References cited in this table are listed at the end of this Supplementary Material and are numbered independently of the main text.

Study	Contribution	Task addressed	Reason-code output
Johnson et al. (2016) Johnson et al. (2016)	MIMIC-III critical-care database	Data resource; not denial prediction	None
Chen & Guestrin (2016) Chen and Guestrin (2016)	XGBoost gradient-boosting system	General tabular learning	None
Shickel et al. (2018) Shickel et al. (2017)	Survey of deep learning for EHR data	Review	None
Lee et al. (2020) Lee et al. (2020)	Clinical predictive model deployment in an EHR	Deployment methodology	None
Talwar et al. (2023) Talwar et al. (2023)	Meta-analysis of readmission prediction	Hospital readmission	None
Sadr et al. (2025) Sadr et al. (2025)	Review of deep learning for healthcare	Review	None
Zhou et al. (2019) Zhou et al. (2019)	Survey of multi-modality image segmentation	Medical imaging	None
Al-Nafjan et al. (2025) Al-Nafjan et al. (2025)	Systematic review of deep learning for outcomes	Review	None
Lahlou et al. (2021) Lahlou et al. (2021)	Transformer-based Medicare claim encoder	Health-risk prediction from claims	None
Hasan (2022) Hasan (2022)	Graph neural network over claim networks	Fraudulent claim detection	None
Kafila et al. (2025) ?	Hybrid ensemble for health-insurance prediction	Insurance outcome forecasting	None
PF-CDM (this work)	Two-stage Random Forest with business rules and SHAP attribution	Denial prediction and reason coding	Top-1 and Top-3 over 67 codes

Table S3. Adjudication-lag sweep. Each cell reports the Pearson correlation between the smoothed prior-denial rate of the entity and the outcome of the index line, computed on the training partition, as the history cut-off is moved further back. A correlation that falls as the cut-off recedes indicates that the shorter cut-off was borrowing outcomes the biller could not have known.

Cut-off (days before the index claim)	Provider history	Patient history
0	0.3244	0.6346
30	0.1775	0.3693
90 (used for provider)	0.1024	0.2686
180	0.1028	0.1678
365 (used for patient)	0.1346	0.1001

Table S4. Stage 1 hyper-parameter search space and selected values. Search used successive halving over 36 candidates on a grouped 30% subsample of training claims; the selected configuration was refitted on all 539,266 training lines with 600 trees.

Parameter	Search space	Selected
n_estimators	150, 250, 400 (600 at refit)	600
max_depth	None, 12, 20, 30	30
min_samples_leaf	1, 2, 5, 10, 20	1
min_samples_split	2, 5, 10, 20	5
max_features	sqrt, log2, 0.3, 0.5	sqrt
class_weight	None, balanced, balanced_subsample	balanced_subsample
SMOTE sampling_strategy	0.5, 0.7, 1.0	0.7
SMOTE k_neighbors	3, 5, 7	3

Table S5. Ablation of the imbalance treatment. Both variants use a 300-tree forest, so their PR-AUC differs slightly from the 600-tree model of Table 1 of the main text. SMOTE is applied within training folds only in both cases.

Variant	PR-AUC	ROC-AUC	Recall	Brier	Fit (min)
SMOTE within training folds	0.7781	0.8652	0.7545	0.1452	11.6
No sampler, balanced subsample weighting	0.7783	0.8639	0.7605	0.1401	6.2
Difference in average precision: −0.0002, 95% CI −0.0009 to +0.0006					

Table S6. Performance over cumulative windows of the held-out period. Prevalence is reported because PR-AUC depends on it; ROC-AUC does not and is given for contrast.

Window	Lines	Prevalence	PR-AUC	ROC-AUC
First month	21,660	0.4477	0.8811	0.8999
First 3 months	57,347	0.3971	0.8311	0.8741
First 6 months	99,280	0.3632	0.7988	0.8662
Full 12 months	139,615	0.3302	0.7770	0.8648

Table S7. Reason-code accuracy as a function of the label space. "Restricted" limits the candidate set to the N most frequent codes and renormalises; "full ranking" retains all 67 candidates and evaluates only lines whose true code is among the N. Both exclude the pooled residual class, which is why the final row differs slightly from the headline figures.

	Restricted		Full ranking			
Label space	Top-1	Top-3	Top-1	Top-3	Lines	% denials
3 codes	0.708	1.000	0.200	0.555	12,866	27.9
5 codes	0.584	0.918	0.238	0.570	18,053	39.2
10 codes	0.363	0.714	0.241	0.521	26,597	57.7
13 codes	0.342	0.661	0.242	0.511	30,050	65.2
20 codes	0.318	0.599	0.261	0.513	36,033	78.2
30 codes	0.301	0.559	0.271	0.511	40,788	88.5
50 codes	0.281	0.522	0.272	0.504	44,235	96.0
67 codes	0.277	0.511	0.272	0.502	44,744	97.1

Table S8. Scrubber design specification and implementation status. "Deployed" means present in the released application; "pipeline" means implemented in the preprocessing used for this study but not exposed in the application; "specified" means designed and not yet built.

Capability	Specification	Status	Source
Format checks	Mandatory fields populated; claim structurally complete	Deployed	Payer submission rules
Field verification	Provider identifiers, payer and patient fields present and well formed	Deployed	Payer submission rules
Schema validation	Batch files carry all 19 required columns	Deployed	Internal
Code validation	Diagnosis and procedure codes valid against ICD-10 and CPT/HCPCS	Pipeline	CMS code sets
Payer normalisation	Payer names standardised to a controlled vocabulary	Pipeline	Internal
Duplicate detection	Repeat submissions identified and flagged	Pipeline	NCCI
Claim format validation	HIPAA 837 and UB-04 conformance	Specified	HIPAA transaction standards
Modifier checks	Modifier use and combinations validated	Specified	CMS coding guidelines
Consistency checks	Diagnosis and procedure codes mutually appropriate	Specified	NCCI edits
Medical necessity	Services cross-referenced against LCDs and NCDs	Specified	CMS coverage determinations
Coverage validation	Patient eligibility and benefits verified against payer policy	Specified	Payer policy
Rule management	Custom rules per payer contract, with versioning	Specified	Internal
Data refresh	Scheduled updates from CMS and NCCI sources	Specified	CMS, NCCI

Table S9. Validation layer as implemented in the released application. These checks govern whether a submission can be scored; none anticipates a reason code.

ID	Scope	Check	Note
V1	Manual entry	Procedure code present	
V2	Manual entry	Billed charge greater than zero	Rejects valid zero-charge reporting codes; under revision
V3	Manual entry	Units at least one	
V4	Manual entry	Drug code present	Rejects all non-drug lines; under revision
V5	Manual entry	Service date precedes claim creation date, calendar-valid	
V6	Batch upload	All 19 required columns present	File rejected otherwise
P1	Pipeline	Procedure codes normalised to five characters	
P2	Pipeline	Diagnosis codes validated against the ICD-10 pattern	Non-conforming values retained and flagged
P3	Pipeline	Payer names standardised	
P4	Pipeline	Exact duplicate lines identified and collapsed	33.9% of the extract
T1	Preprocessing	Numeric coercion, failures set to zero	
T2	Preprocessing	Unseen categorical values mapped to a baseline class	Prevents failure on unfamiliar codes
T3	Preprocessing	Payer type expanded to four binary indicators	

Table S10. Candidate reason-code rules, measured on the held-out partition. These are not deployed; they are reported to establish whether such a layer is worth constructing. Provenance: P = payer policy, N = national coding edit.

ID	Category	Condition	CARC	Prov.	Lines	PPV
R01	Timeliness	Submission lag exceeds 365 days	29	P	3,601	0.416
R02	Documentation	Referring provider identifier absent	16	P	21,035	0.139
R03	Duplicate	Same procedure and charge more than once on the claim	18	N	24,906	0.047

Table S11. Stage 1 performance by subgroup on the held-out partition. Subgroups with fewer than 200 lines, or with a single outcome class, are omitted. Prevalence is the denial rate within the subgroup and FPR the false-positive rate at the operating threshold of 0.469. Provider specialties are identified by taxonomy code. Lines on which an attribute is not recorded are retained as their own group rather than discarded, since discarding them would change the composition of the partition.

Subgroup	Lines	Prev.	PR-AUC	ROC-AUC	Recall	Prec.	FPR
Payer type							
Commercial	57,295	0.370	0.801	0.874	0.770	0.710	0.185
BCBS	38,942	0.274	0.760	0.887	0.764	0.667	0.144
Medicare	34,582	0.259	0.652	0.793	0.673	0.479	0.256
Medicaid	8,796	0.596	0.938	0.924	0.823	0.942	0.075
Provider specialty							
207RC0000X	27,551	0.149	0.736	0.918	0.703	0.651	0.066
208100000X	27,351	0.349	0.809	0.897	0.744	0.767	0.121
291U00000X	23,542	0.475	0.820	0.801	0.704	0.748	0.214
207R00000X	18,721	0.182	0.681	0.860	0.591	0.641	0.074
225100000X	16,843	0.506	0.788	0.797	0.823	0.685	0.388
207Q00000X	6,813	0.329	0.700	0.829	0.760	0.584	0.265
363A00000X	3,584	0.403	0.799	0.832	0.877	0.578	0.432
363LF0000X	3,574	0.419	0.791	0.823	0.928	0.532	0.589
207RI0011X	3,197	0.625	0.879	0.853	0.950	0.743	0.547
363AM0700X	3,088	0.213	0.648	0.771	0.790	0.284	0.537
101Y00000X	1,468	0.156	0.575	0.835	0.795	0.319	0.314
207RG0300X	984	0.244	0.443	0.613	0.650	0.281	0.538
207RC0001X	829	0.597	0.843	0.783	0.921	0.682	0.638
363LP0808X	637	0.104	0.210	0.599	0.651	0.140	0.462
193200000X	458	0.072	0.255	0.768	0.303	0.159	0.125
363L00000X	394	0.360	0.702	0.724	0.690	0.462	0.452
363LA2100X	309	0.599	0.871	0.881	0.789	0.864	0.185
Patient state							
not recorded	69,095	0.246	0.742	0.877	0.732	0.630	0.140
MI	47,176	0.397	0.798	0.869	0.778	0.722	0.197
IL	21,499	0.421	0.811	0.800	0.759	0.612	0.350
FL	892	0.821	0.880	0.587	0.814	0.822	0.806
NJ	357	0.611	0.690	0.604	0.560	0.709	0.360
Age band							
45-64	51,883	0.338	0.782	0.876	0.785	0.673	0.195
75+	34,180	0.291	0.737	0.840	0.676	0.609	0.178
65-74	31,486	0.306	0.751	0.861	0.760	0.620	0.205
18-44	19,430	0.431	0.853	0.882	0.791	0.768	0.181
0-17	2,636	0.234	0.758	0.809	0.679	0.850	0.037
Sex							
not recorded	68,986	0.418	0.799	0.840	0.771	0.687	0.253
Female	35,520	0.247	0.733	0.866	0.719	0.608	0.152
Male	35,080	0.240	0.751	0.890	0.742	0.651	0.126

Table S12. Parity measures on the largest contrast within each attribute. The equal opportunity difference is the difference in recall between the two groups and the FPR ratio the ratio of their false-positive rates; values near zero and one respectively indicate parity.

Attribute	Group A	Lines A	Group B	Lines B	Equal opp. diff.	FPR ratio
Payer type	Commercial	57,295	BCBS	38,942	+0.0068	1.285
Provider specialty	207RC0000X	27,551	208100000X	27,351	-0.0416	0.549
Patient state	not recorded	69,095	MI	47,176	-0.0461	0.713
Age band	45-64	51,883	75+	34,180	+0.1085	1.090
Sex	not recorded	68,986	Female	35,520	+0.0516	1.663

2 SUPPLEMENTARY FIGURES

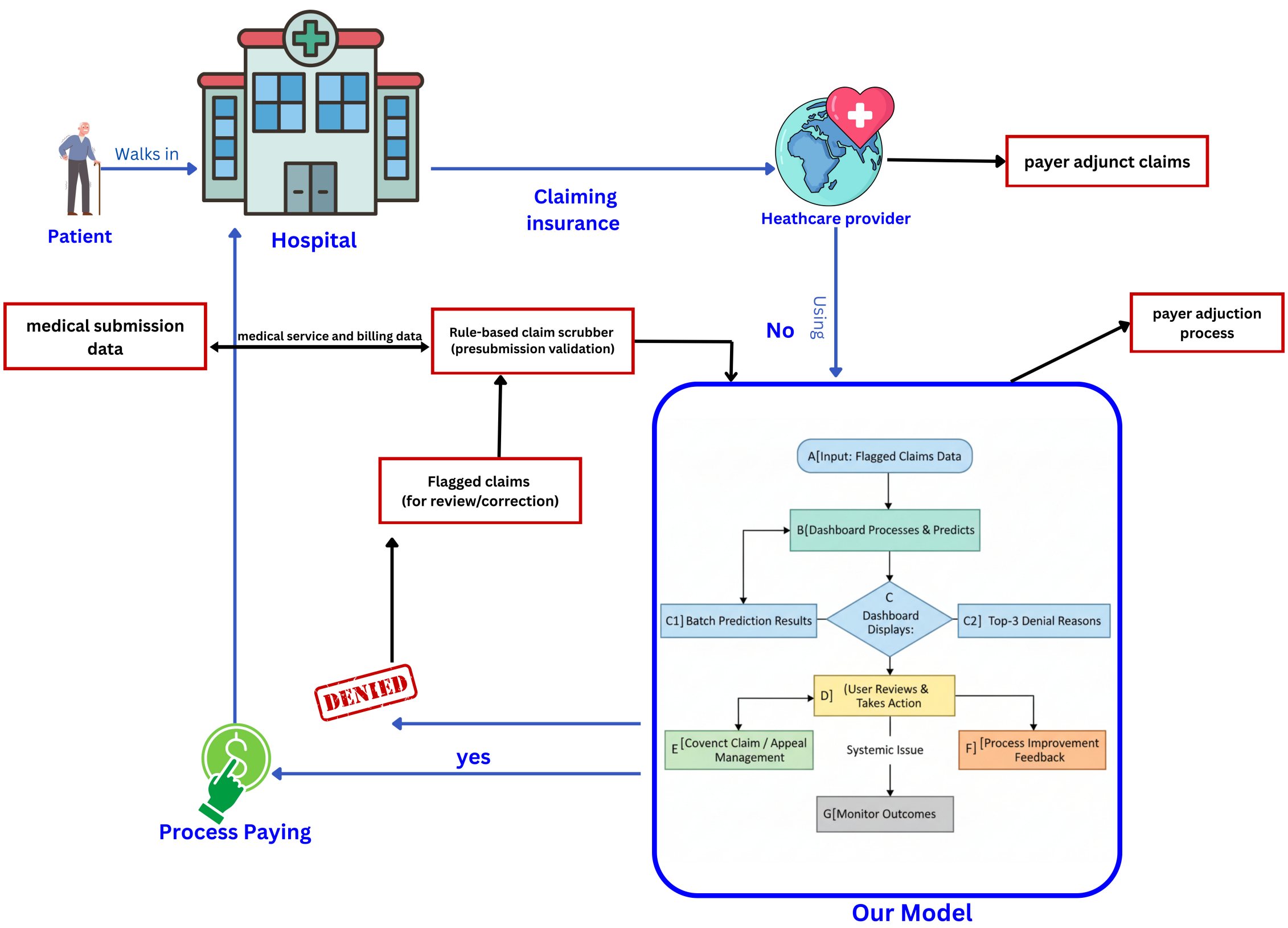

Figure S1. Workflow Diagram of the Classification Process

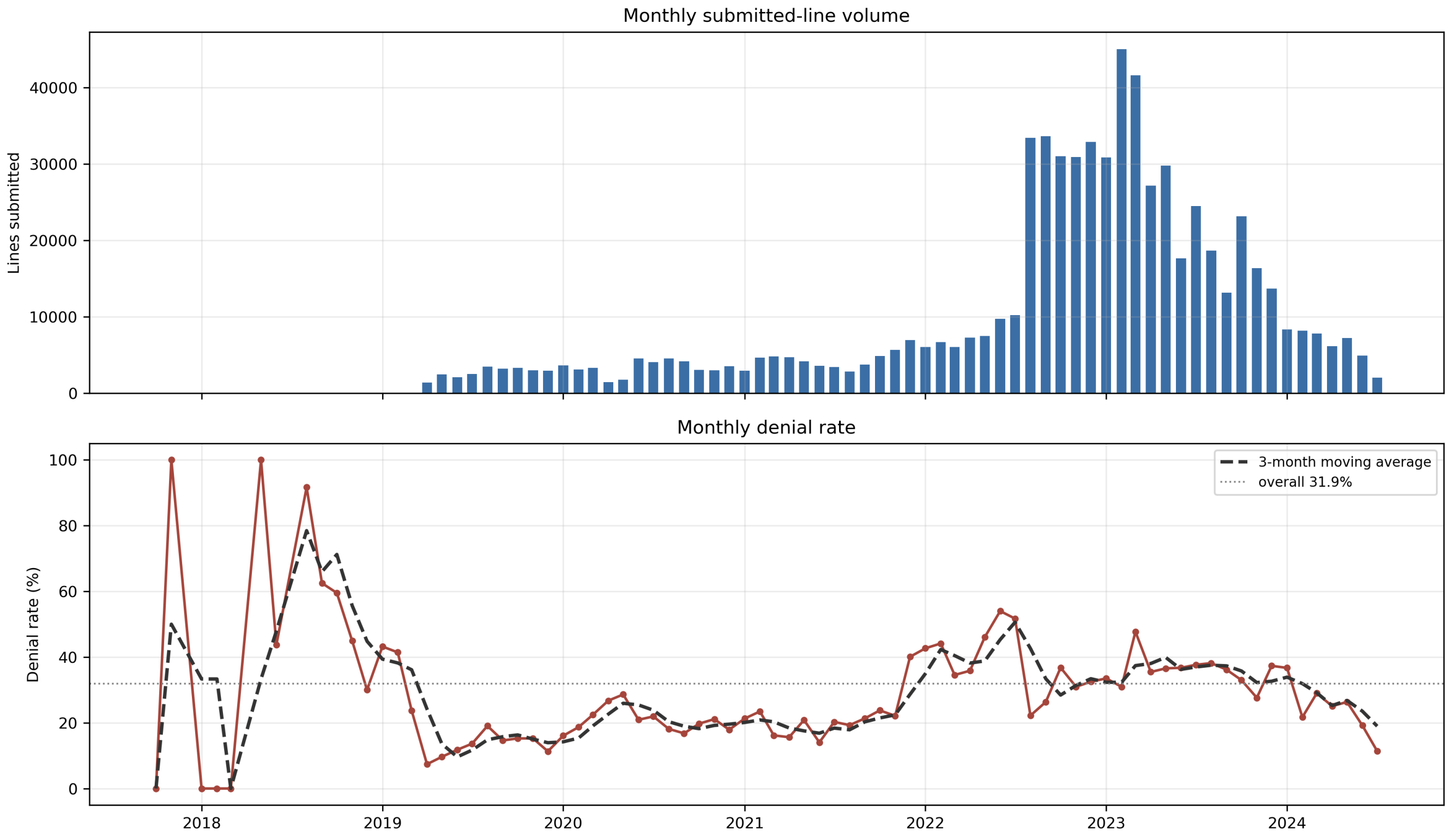

Figure S2. Monthly submitted-line volume and monthly denial rate with a three-month moving average, 2017–2024. Volume rises from 2019 and peaks in 2023; the denial rate varies between roughly 20 and 45% across the period.

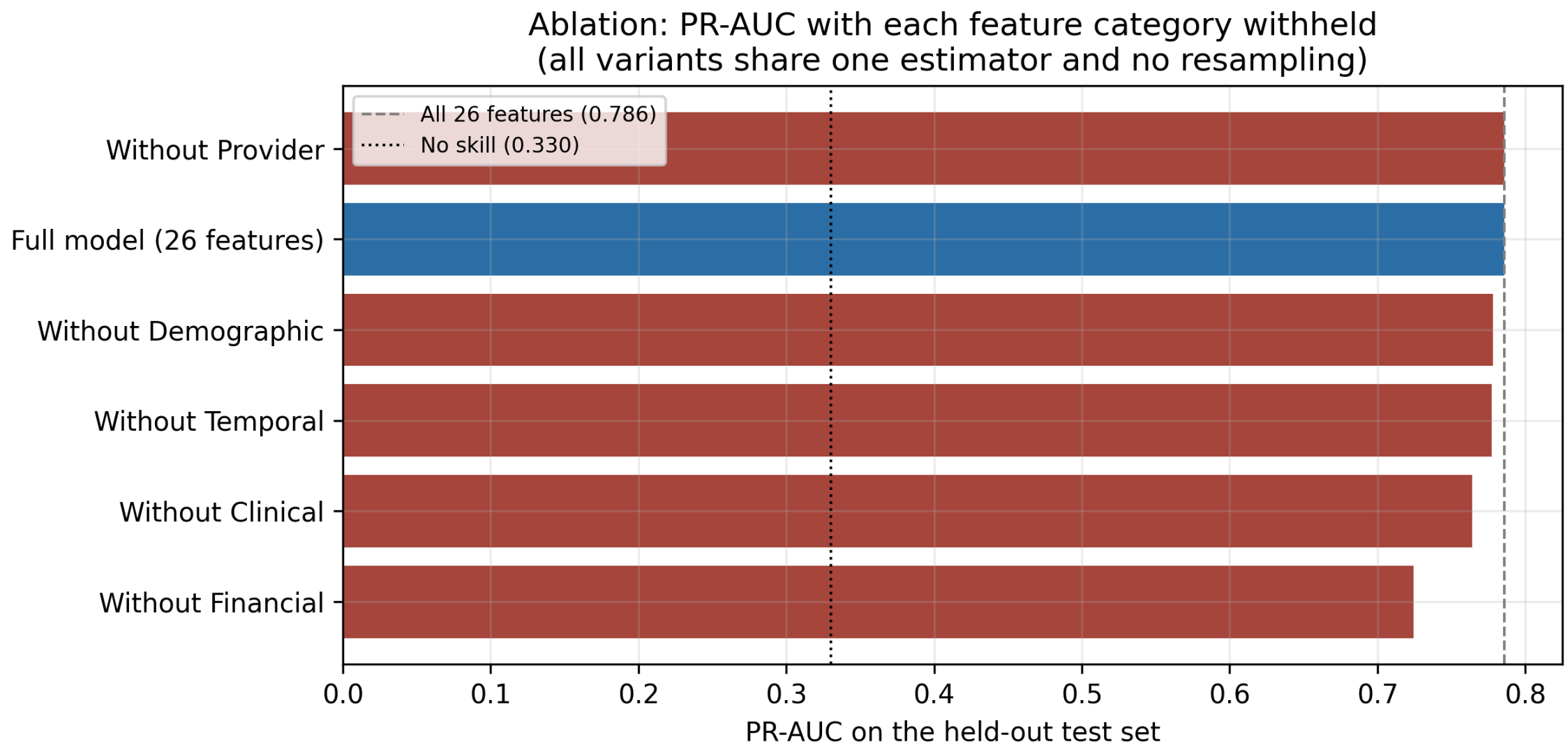

Figure S3. Feature-category ablation on PR-AUC, all variants sharing one estimator and no resampling, with the no-skill line drawn. Using a matched reference is necessary: comparing unsampled variants against a differently configured model can make a category appear to improve performance when withheld.

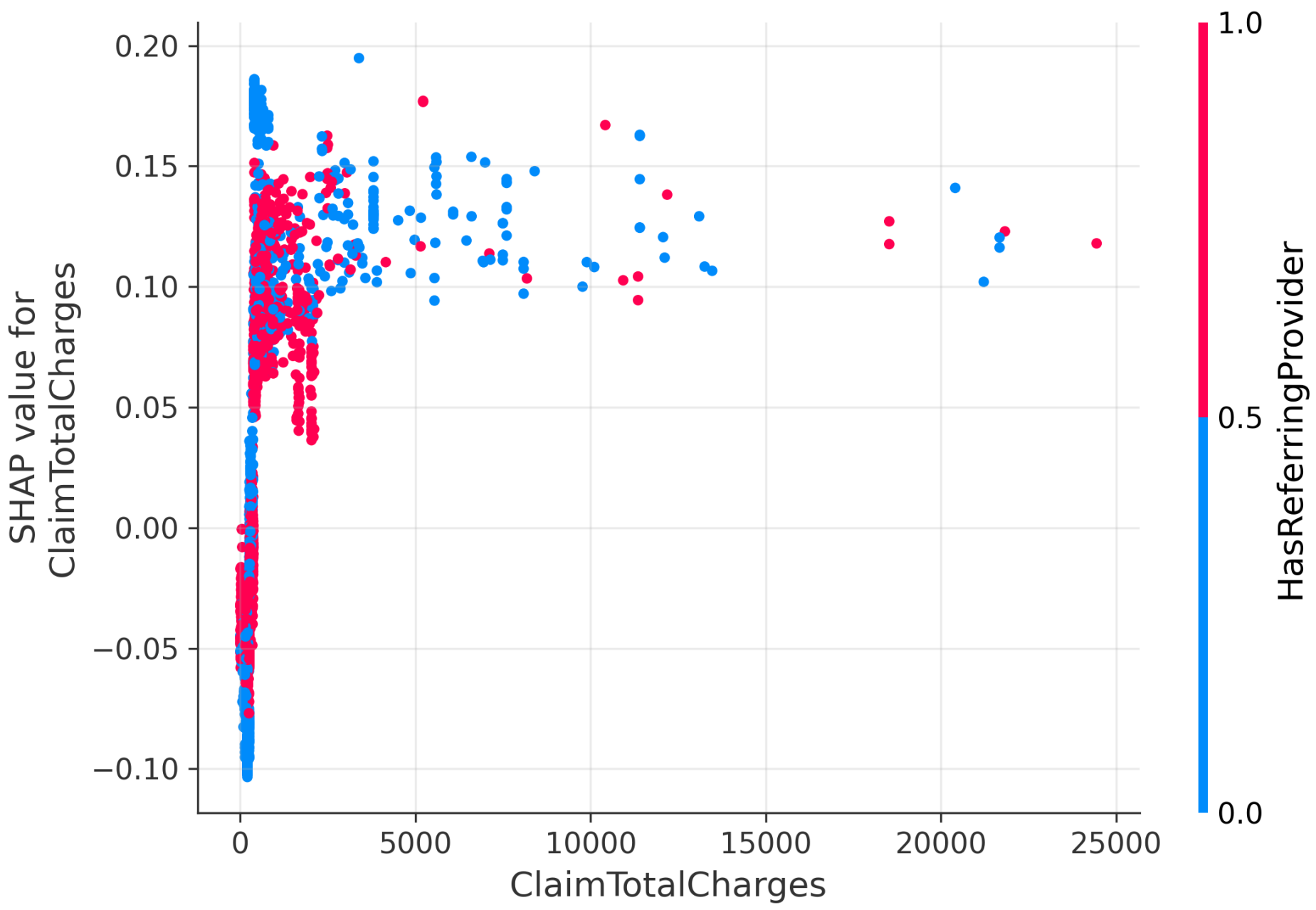

Figure S4. SHAP dependence for the total charge of the claim, the strongest single contributor.

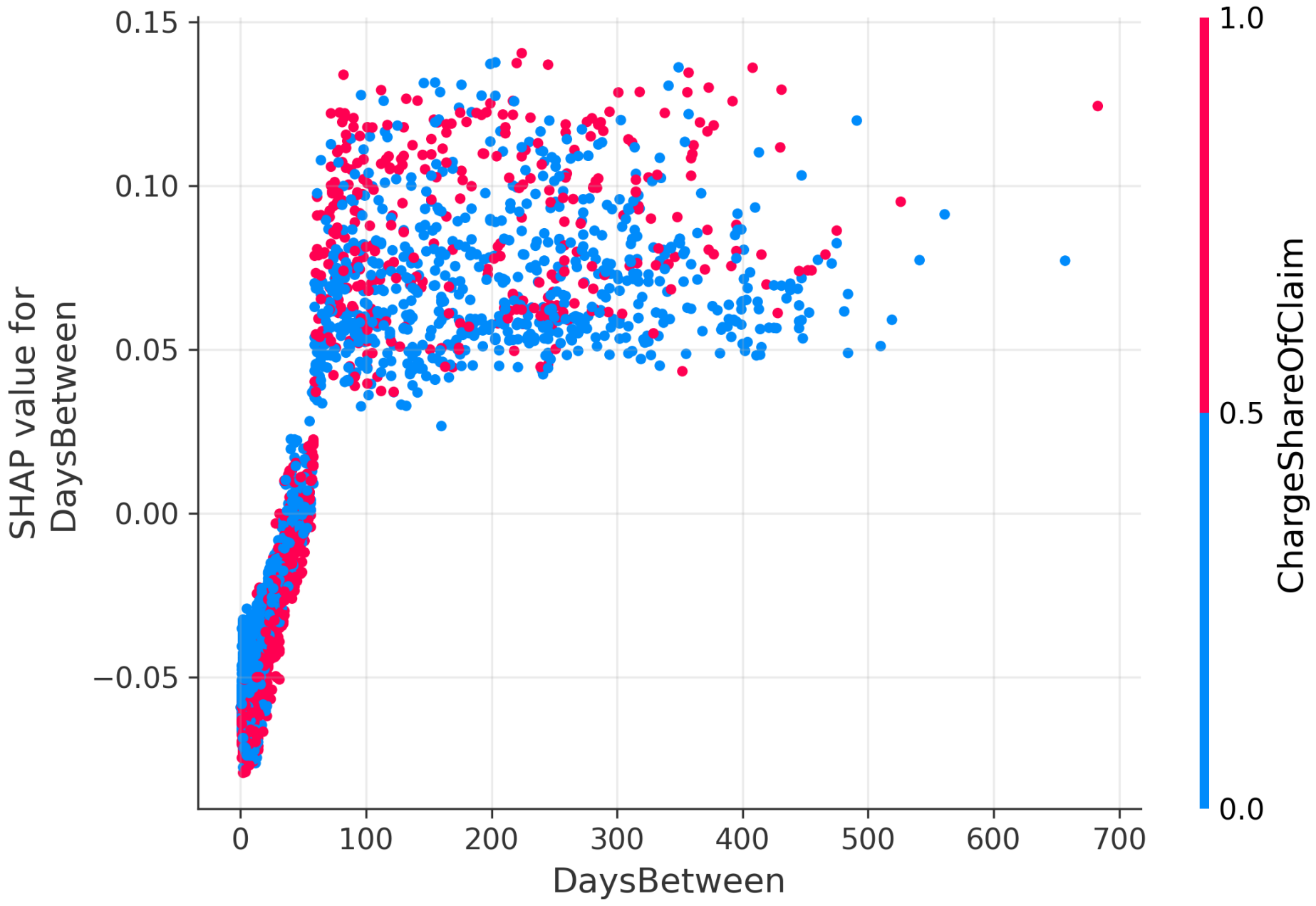

Figure S5. SHAP dependence for the submission lag, the second strongest contributor. Attribution rises steeply beyond roughly 30 days and again beyond 90, which is consistent with the timely-filing windows operated by the payer and with the two lag indicators retained by the feature selection.

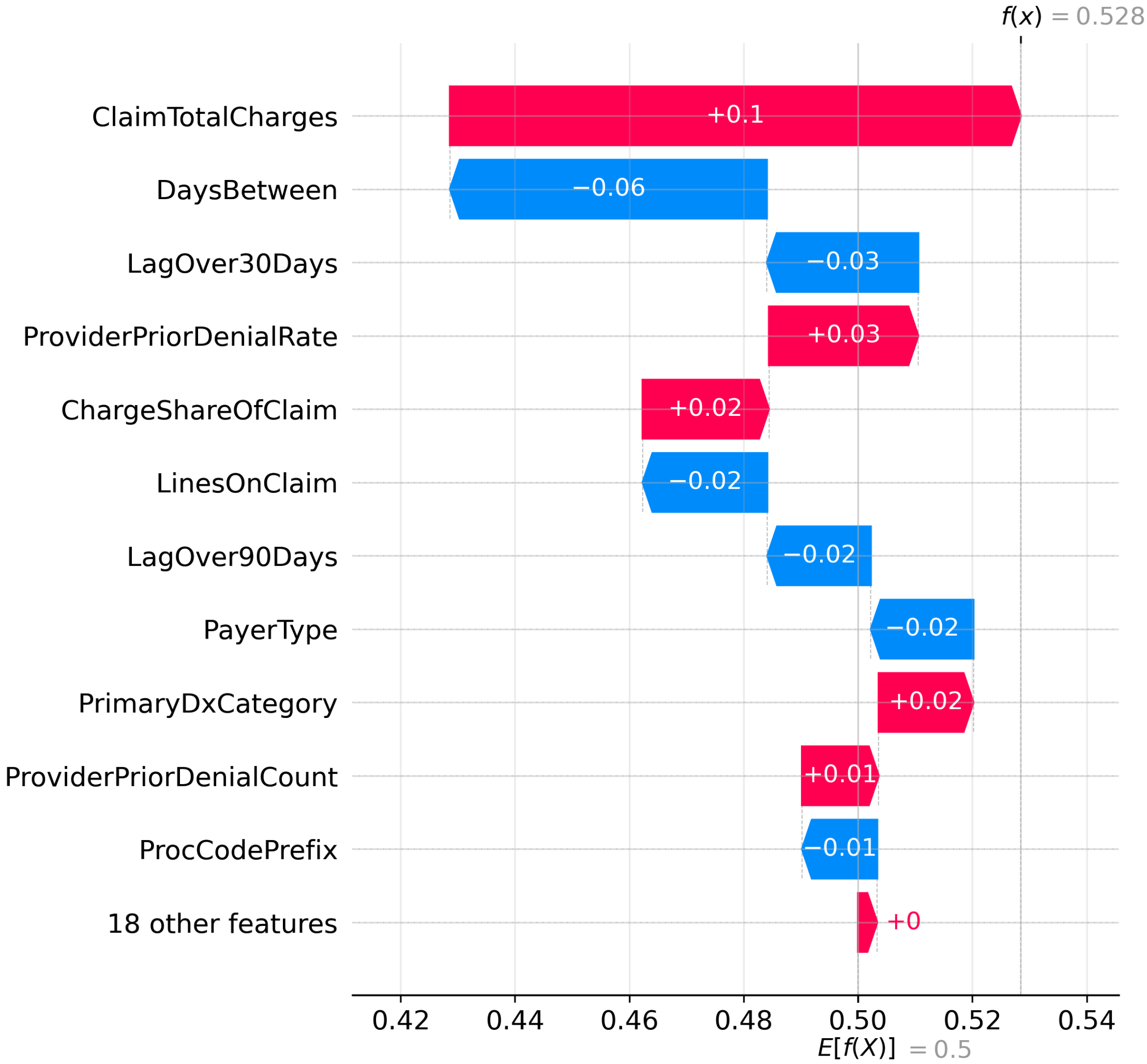

Figure S6. Local explanation for a true positive: a line the model flagged that was in fact denied. This is the case the system is intended to catch, and the attribution shows which claim characteristics drove the prediction.

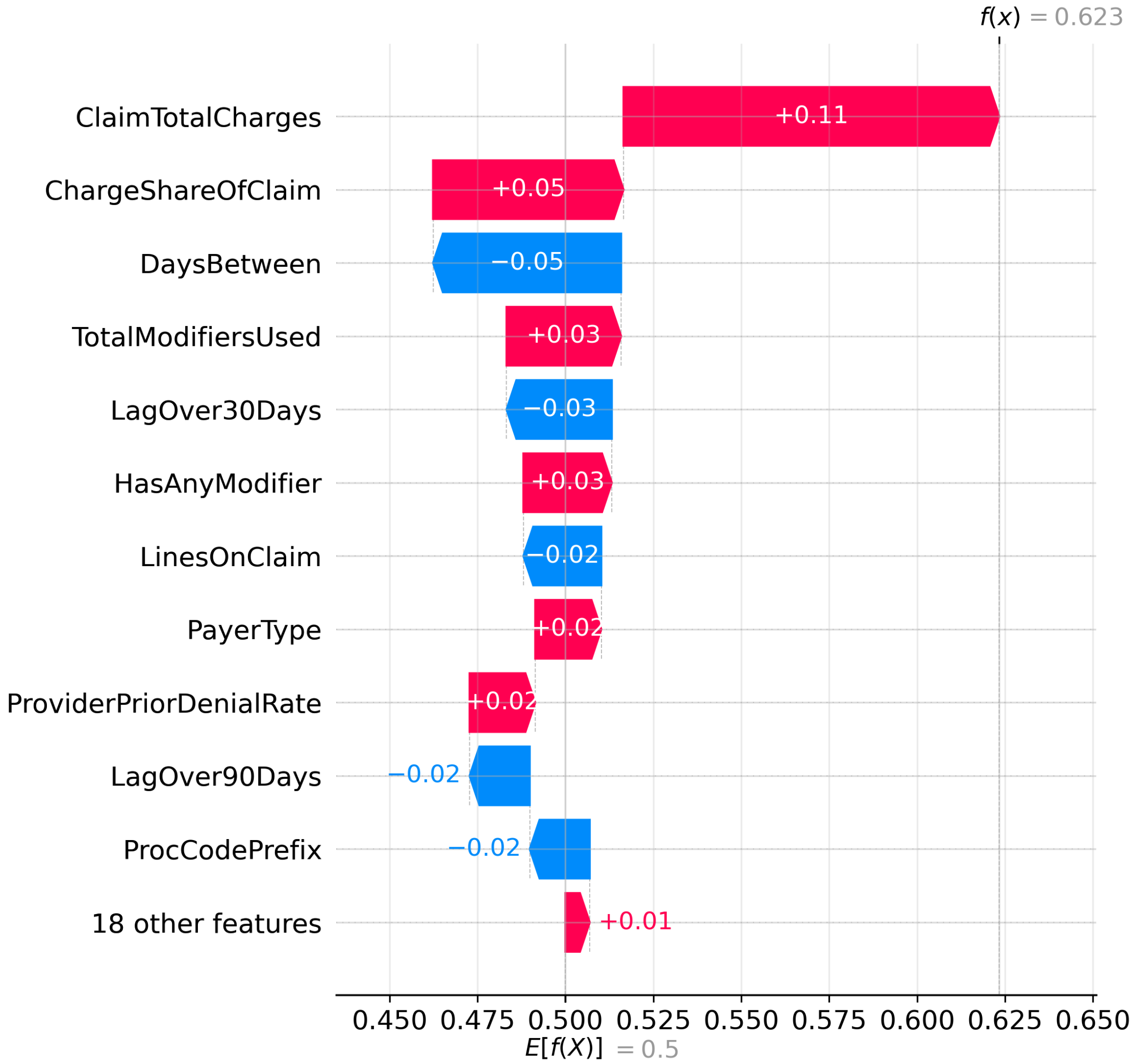

Figure S7. Local explanation for a false positive: a line the model flagged that was in fact paid. This is the case a billing team carries the cost of, since it generates an unnecessary review.

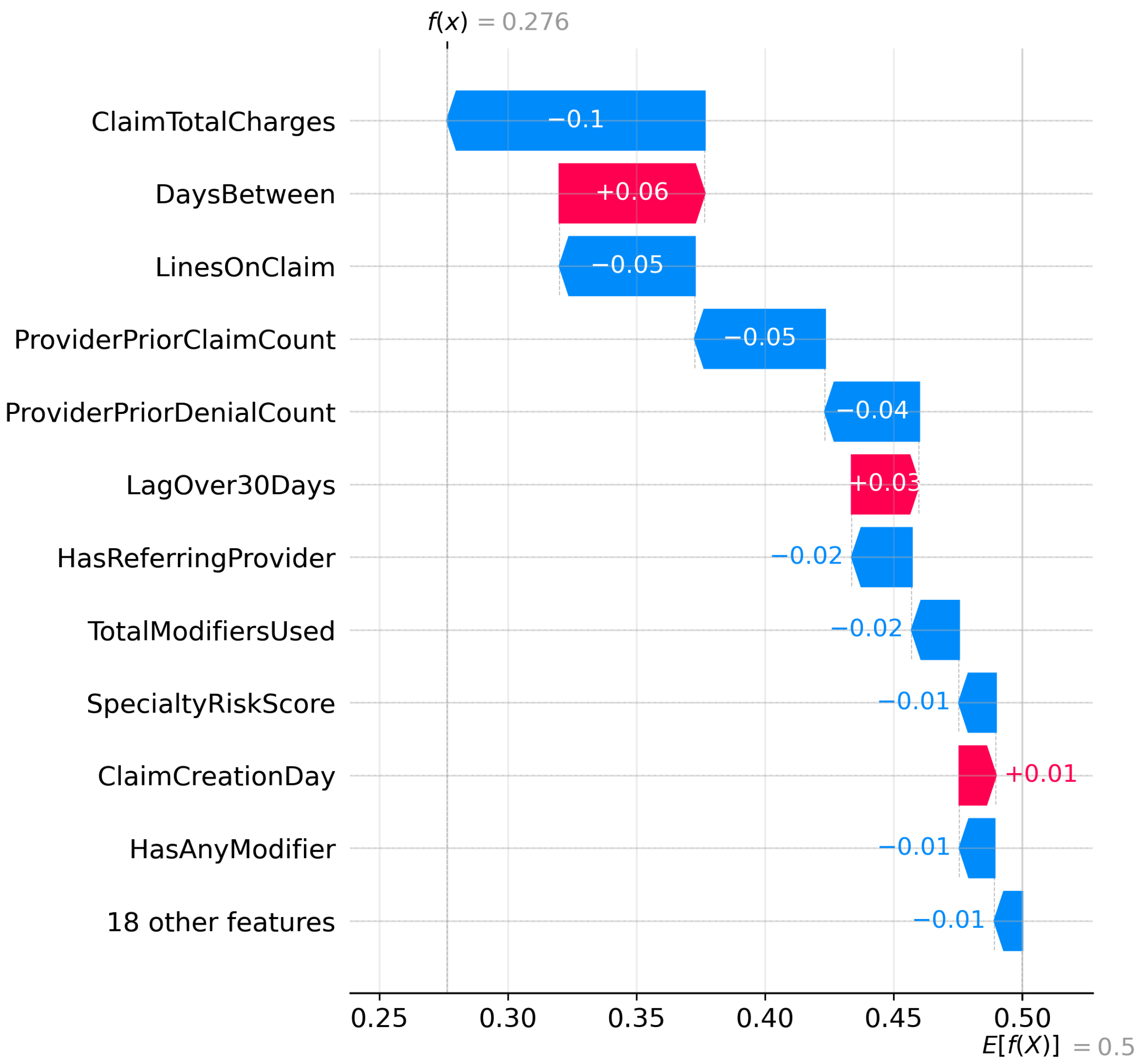

Figure S8. Local explanation for a false negative: a denied line the model did not flag, and therefore the case that reaches the payer unreviewed. The attribution shows the model weighting the small charge more heavily than the extreme submission lag, which is the error mode discussed in Section 5 of the main text.

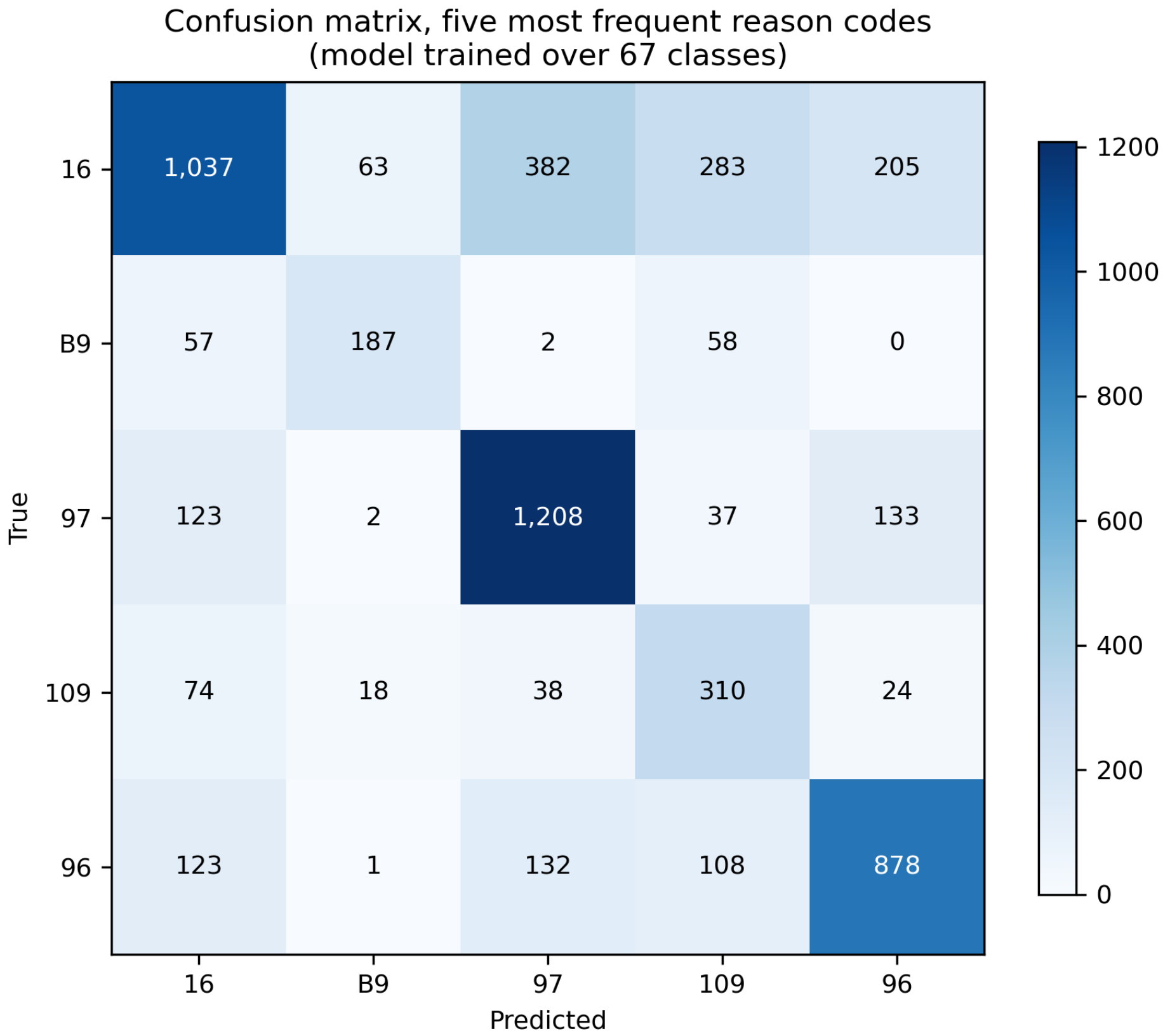

Figure S9. Confusion matrix over the five most frequent reason codes, for a model trained over the full 67-class label space.

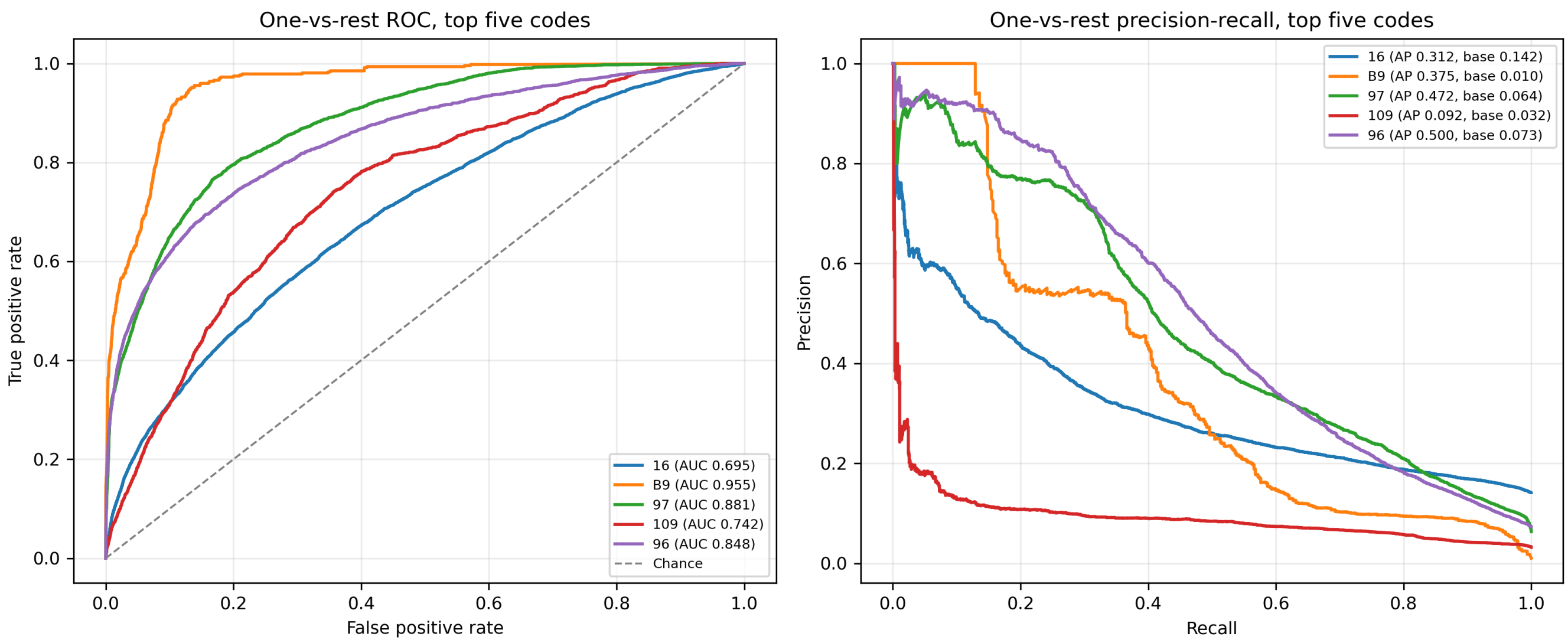

Figure S10. One-vs-rest ROC and precision-recall curves for the five most frequent codes. Each precision-recall legend states its own no-skill baseline, since the codes differ in prevalence.

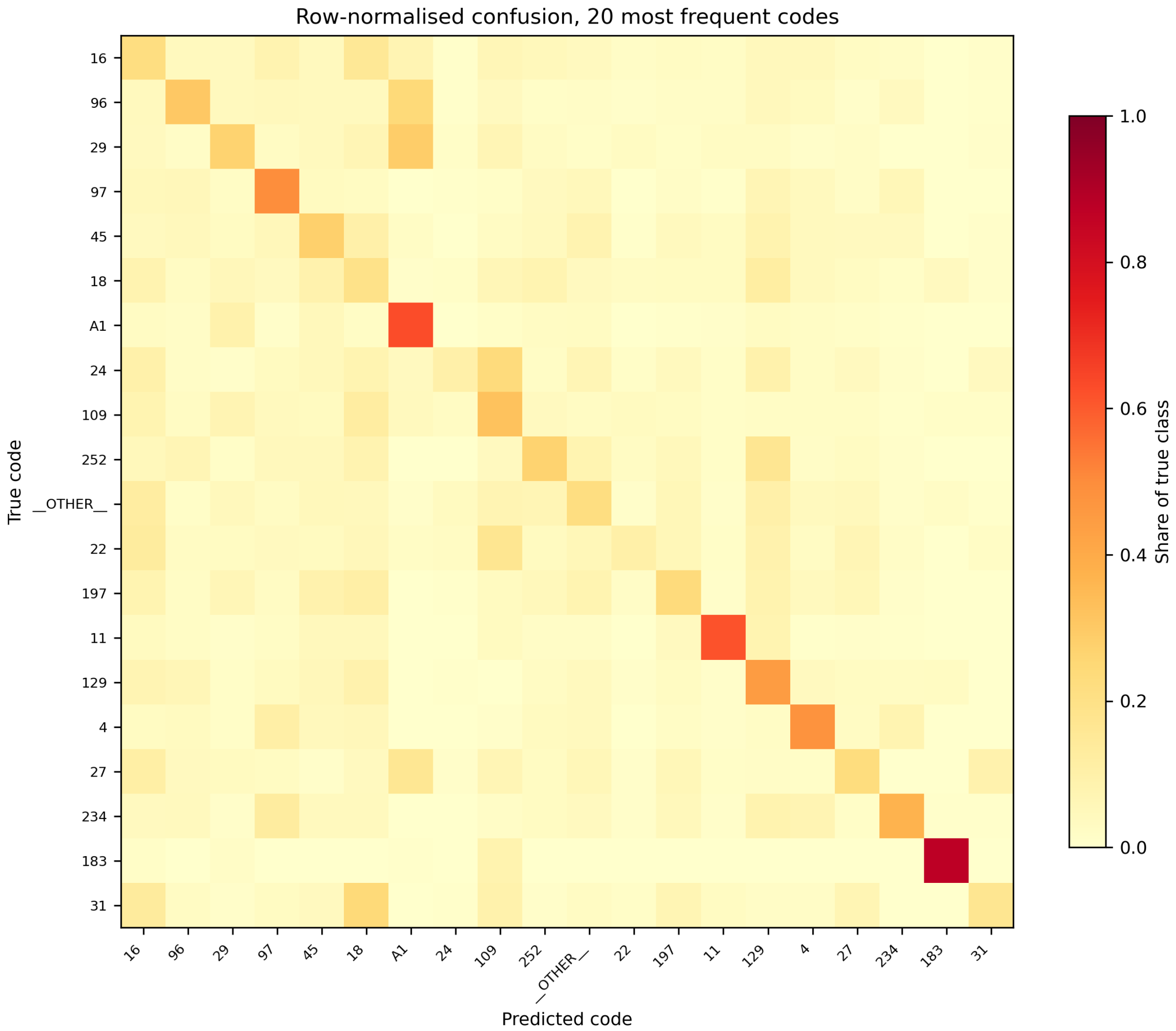

Figure S11. Row-normalised confusion over the twenty most frequent reason codes.

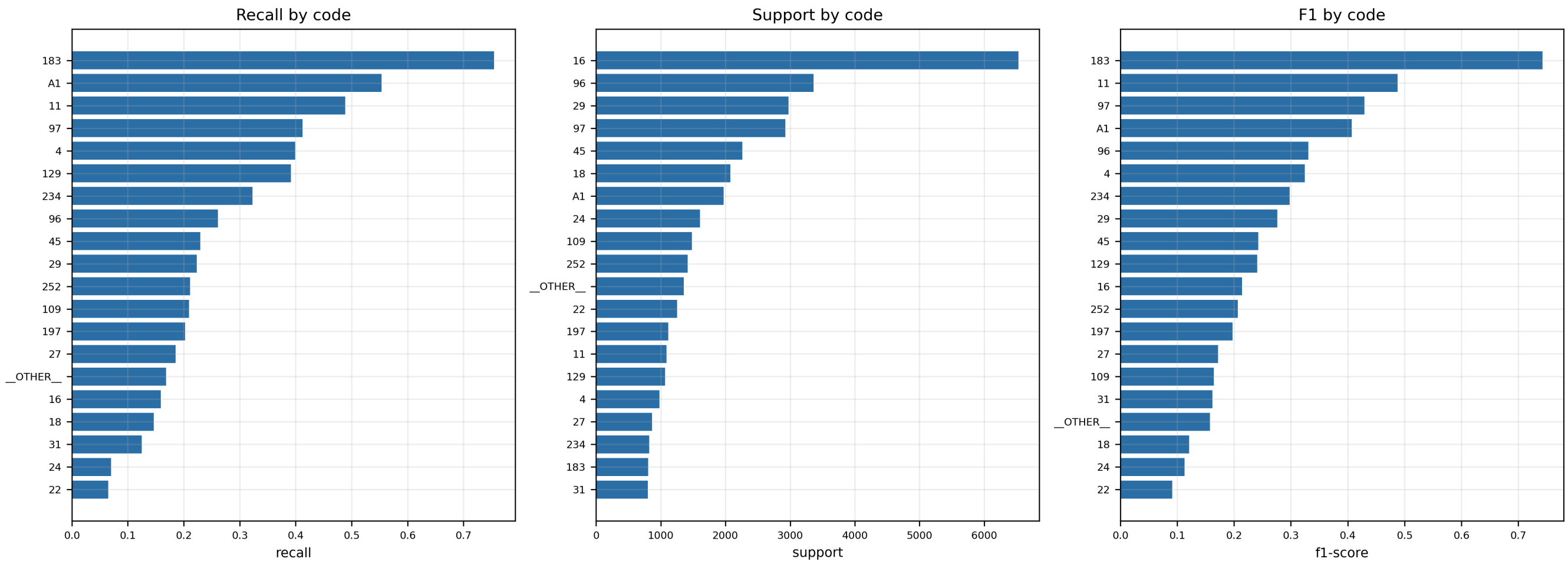

Figure S12. Recall, support and F_1 for the twenty most frequent reason codes. All three panels are computed over the 67-class label space.

REFERENCES

Johnson AE, Pollard TJ, Shen L, Lehman LWH, Feng M, Ghassemi M, et al. Mimic-iii, a freely accessible critical care database. Scientific Data 3 (2016) 1–9. doi:10.1038/sdata.2016.35.

Chen T, Guestrin C. Xgboost: A scalable tree boosting system. Proceedings of the 22nd ACM SIGKDD International Conference on Knowledge Discovery and Data Mining (2016), 785–794. doi:10.1145/2939672.2939785.

Shickel B, Tighe PJ, Bihorac A, Rashidi P. Deep ehr: a survey of recent advances in deep learning techniques for electronic health record analysis. IEEE Journal of Biomedical and Health Informatics 22 (2017) 1589–1604. doi:10.1109/JBHI.2017.2767063.

Lee TC, Shah NU, Haack A, Baxter SL. Clinical implementation of predictive models embedded within electronic health record systems: A systematic review. Informatics 7 (2020) 25. doi:10.3390/informatics7030025.

Talwar A, Lopez-Olivo MA, Huang Y, Ying L, Aparasu RR. Performance of advanced machine learning algorithms over logistic regression in predicting hospital readmissions: A meta-analysis. Exploratory Research in Clinical and Social Pharmacy 11 (2023) 100317. doi:10.1016/j.rcsop.2023.100317.

Sadr H, Nazari M, Khodaverdian Z, Farzan R, Yousefzadeh-Chabok S, Ashoobi MT, et al. Unveiling the potential of artificial intelligence in revolutionizing disease diagnosis and prediction: A comprehensive review of machine learning and deep learning approaches. European Journal of Medical Research 30 (2025) 418.

Zhou T, Ruan S, Canu S. A review: Deep learning for medical image segmentation using multi-modality fusion. Array 3 (2019) 100004. doi:10.1016/j.array.2019.100004.

Al-Nafjan A, Aljuhani A, Alshebel A, Alharbi A, Alshehri A. Artificial intelligence in predictive healthcare: A systematic review. Journal of Clinical Medicine 14 (2025) 6752. doi:10.3390/jcm14196752.

Lahlou C, Crayton A, Trier C, Willett E. Explainable health risk predictor with transformer-based medicare claim encoder. arXiv preprint arXiv:2105.09428 (2021). doi:10.48550/arXiv.2105.09428.

Hasan MT. Graph neural network models for detecting fraudulent insurance claims in healthcare systems. American Journal of Advanced Technology and Engineering Solutions 2 (2022) 88–109. doi:10.63125/r5vsmv21.
